# Unveiling the Mechanism of Deprotonation and Proton Transfer of DNA Polymerase Catalysis via Single‐Molecule Conductance

**DOI:** 10.1002/advs.202408112

**Published:** 2024-11-21

**Authors:** Lihua Zhao, Yang Xu, Zhiheng Yang, Wenzhe Liu, Shichao Zhong, Jingwei Bai, Xuefeng Guo

**Affiliations:** ^1^ Beijing National Laboratory for Molecular Sciences National Biomedical Imaging Center College of Chemistry and Molecular Engineering Peking University 292 Chengfu Road, Haidian District Beijing 100871 P. R. China; ^2^ School of Pharmaceutical Sciences Tsinghua University Beijing 100093 P. R. China; ^3^ Center of Single‐Molecule Sciences Institute of Modern Optics Frontiers Science Center for New Organic Matter College of Electronic Information and Optical Engineering Nankai University 38 Tongyan Road, Jinnan District Tianjin 300350 P. R. China

**Keywords:** catalysis mechanism, DNA polymerase, electric field, GMG biosensor, hPol β protein, single‐molecule level

## Abstract

DNA polymerases (Pols) play important roles in the transmission of genetic information. Although the function and (de)regulation of Pols are linked to many human diseases, the key mechanism of 3′‐OH deprotonation and the PP_i_ formation are not totally clear. In this work, a method is presented to detect the full catalytic cycle of human Pol (hPol *β*) in graphene‐molecule‐graphene single‐molecule junctions. Real‐time in situ monitoring successfully revealed the spatial and temporal properties of the open and closed conformation states of hPol *β*, distinguishing the reaction states in the Pols catalytic cycle and unveiling 3′‐OH deprotonation and pyrophosphate (PP_i_) formation mechanism of hPol *β*. Proton inventory experiment demonstrated that the rate‐limiting step of PP_i_ formation is deprotonation, which occurs before a reverse conformational change. Additionally, by detecting the acidity (p*K*_a_), it is found that Mg_A_‐bound OH^−^ acted as a general base and activated the nucleophile of 3′‐OH, and that acidic residue D190 or D192 coordinated with Mg_B_ as a proton donor to PP_i_. This work provides useful insights into a fundamental chemical reaction that impacts genome synthesis efficiency and Pol fidelity, which the discovery of Pol‐targeting drugs and design of artificial Pols for DNA synthetic applications are expected to accelerated.

## Graphical abstract

For the first time, a single‐molecule electrical analysis strategy has been developed to directly decipher the dynamic process of DNA replication. The conductance signals elucidate the key mechanism of 3′‐OH deprotonation and the pyrophosphate (PP_i_) formation. Mg_A_‐bound OH^−^ activates the nucleophile of 3′‐OH, while D190 or D192, coordinated with Mg_B,_ promotes the PPi formation.

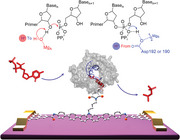

## 1 Introduction

**Figure 1:**
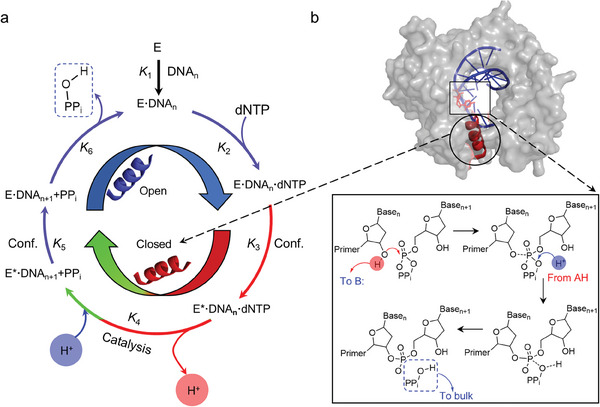
Nucleotide addition catalytic cycle of human Pol *β* (hPol *β*). a) Minimum reaction pathway of Pol showing the key steps in DNA polymerization and the primary accompanying mechanical motion. The blue *α*–helix N indicates the open configuration; the red *α*–helix N indicates the closed configuration; and the green part indicates the uncertainty in the mechanistic of the PP_i_ generation.^[^ ^5b^ ^]^ b) Crystal structure of hPol (PDB ID: 4KLG). The circle marks the *α*–helix N. The blue part is the closed configuration and the red part is the of open configuration of hPol *β*. The box marks the active site. In the chemical step, the 3′‐OH group of the primer terminus (n) is deprotonated, and attacks the P_α_ atom of the incoming nucleotide (n + 1). Then, the pyrophosphate group (PP_i_) takes a proton and is released. B, general base; AH, general acid.

DNA polymerases (Pols) underlie the survival and propagation of life.^[^ ^1^ ^]^ Pols have core roles in the transmission of genetic information, and errors that lead to gene mutations are related to various human diseases.^[^ ^2^ ^]^ The discovery of Pols (including DNA and RNA enzymes) and the study of their catalytic mechanism have been recognized many times by Nobel prizes. For example, the Nobel Prize in Physiology/Medicine 1959 was awarded to Severo Ochoa and Arthur Kornberg “for their discovery of the mechanisms in the biological synthesis of RNA and DNA.”^[^ ^3^ ^]^ Several decades later, the structural and conformational changes, and catalytic mechanisms of Pols were established by various structural and kinetic methods, including time‐resolved X‐ray crystallography,^[^ ^4^ ^]^ as summarized in several recent reviews.^[^ ^5^ ^]^ In each catalytic cycle, a dNTP complementary to the template base is incorporated into DNA. Then, a conformational change of the N‐subdomain (*α*–helix N) from open to closed is triggered to align NTP with the 3′‐OH for nucleophilic attack. Next, a pyrophosphate (PP_i_) group is formed and the reverse conformational change (closed to open) occurs, followed by the release of PP_i_ (**Figure** **1**a).^[^ ^6^ ^]^ However, these structural methods are generally proton invisible and cannot capture fast dynamic events. Despite extensive kinetic studies using the stopped‐flow technique and the dNTP analog dNTPaS,^[^ ^7^ ^]^ the rate‐limiting step in DNA synthesis corresponds to re‐opening, which is limited by the chemical step. Therefore, the non‐covalent binding step occurring during the enzyme's open conformation is easily overwhelmed, although it can impact genome synthesis efficiency and Pol fidelity. Considering the fundamental limitation that only average properties are obtained in experiments on the large ensemble of molecules in bulk experiments, very little information is available on the role of active‐site residues in the chemistry of nucleotidyl transfer. After decades of investigations, it remains uncertain how a 3′‐OH nucleophile is activated (Figure S1, Supporting information)^[^ ^8^ ^]^ and the mechanistic of the PP_i_ formation are still not well understood (Figure 1b),^[^ ^9^ ^]^ and have long been a challenge in this field.

DNA Polymerase *β*, a Pol X family member, is chosen as the model enzyme because of its small size, simple structure (lacking a proofreading activity),^[^ ^10^ ^]^ complete DNA repair mechanism,^[^ ^11^ ^]^ and particularly substantial evidence of a third divalent metal ion observed,^[^ ^12^ ^]^ which is possible to expand DNA replication mechanism. However, there are few reports studying the mechanism of DNA replication catalyzed by Pols. Although hPol *β* have been employed as model enzymes in the study of DNA repair mechanisms,^[^ ^8^, ^11^, ^13^ ^]^ their relatively simple structure also renders them excellent models for investigating DNA replication mechanisms.^[^ ^8^, ^14^ ^]^ In this assay, an extended DNA replication mechanisms.^[^ ^8^, ^14^ ^]^ In this assay, an extended DNA template primer as shown below was used in the experiment:

3′ –ACATTTTGCTGCCGGTCA AAAAA AAAAA AAAAA–5′

5′ –TGTAAAACGACGGCCAGT–3′

At present, the role of metal ion at the A–site (Mg_A_) and B–site (Mg_B_) for DNA polymerization has been established by extensive supporting evidence.^[^ ^15^ ^]^ Mg_A_ is coordinated with three conserved acidic residues (D190, D192, and D256), two of which (D190 and D192) are also coordinated with Mg_B_.^[^ ^14a^ ^]^ Mg_A_ is believed to lower the acidity (p*K*_a_) of the primer 3′‐OH.^[^ ^16^ ^]^ Using quantum mechanics/molecular mechanics (QM/MM) free energy simulations, Matute *et al.* demonstrated that the 3′‐OH nucleophile was activated by proton transfer to a deprotonated Mg_A_‐bound OH^−^.^[^ ^17^ ^]^ But, Alberts et al.^[^ ^8c^ ^]^ proposed a water molecule bound to Mg_A_ as the general base for proton transfer to D190, which behaves as a potential proton donor to PP_i_, based on mixed QM/MM calculations. However, the feasibility of these mechanisms remains to be further validated.

Using a single polymerase molecule as a real‐time observation object (i.e., direct observation of the nucleotide addition cycle) is useful for tracking the kinetics of stepwise reactions. Xie et al. utilizes fluorescence assays to monitor the conformational change in T7 DNA polymerase upon dNTP binding, demonstrating a high selectivity for correct dNTPs.^[^ ^18^ ^]^ By single‐molecule Förster resonance energy transfer, Rueda et al. observed dynamics of E. coli DNA polymerase I during DNA synthesis, revealing a potential novel step in the proofreading process following correct nucleotide incorporation.^[^ ^19^ ^]^ These experimental techniques, which make observations more accurate or enable the observation of phenomena that were previously unobservable, is valuable for exploring the step‐by‐step enzyme cyclic reaction. In recent years, single‐molecule junction techniques, known for their high spatial/temporal resolution and no need for fluorescent labeling, have shown great promise in studying reaction mechanisms. Therefore, employing single‐molecule electrical detection technology to investigate the mechanism of polymerases is expected not only to provide deep insights into the intrinsic molecular mechanism of DNA polymerization catalyzed by polymerases, but also to broaden the research method for studying biological systems. Considering the intrinsic speed, the covert active site, and the big size of DNA Pols, several technical challenges must be met simultaneously: i) the rate of DNA synthesis catalyzed by each DNA Pol exhibits random fluctuations; therefore, while undergoing template‐directed synthesis, each DNA Pol needs to be observed separately; ii) the active site is covert, and therefore the testing site must be sufficiently close to the active site of the Pol being detected; iii) considering the large size of the DNA Pol, a single fixed point is required to maintain testing repeatability; and iv) an instrument is required that can faithfully provide real‐time detection and sensitivity toward structural transformations.

Here, we present such an approach to realize single‐molecule real‐time electrical monitoring of the catalytic cycle of hPol *β*. A single Pol molecule was assembled into a molecular nanocircuit to track the current change for each nucleotide addition. Graphene‐molecule‐graphene (GMG) single‐molecule junctions were chosen as the molecular nanocircuit because the geometric structural and chemical reactions of a molecule are known to be closely related to their conductance.^[^ ^20^ ^]^ The mutation point W325C was selected near the active site^[^ ^21^ ^]^ to achieve a high level of molecular conductance sensitivity. Moreover, by a special biorthogonal reaction, the maleimide functional center of the molecular bridge reacted with the only sulfhydryl group (C325) on the Pol surface through a Michael addition,^[^ ^22^ ^]^ ensuring the testing repeatability of dynamic electrical tracing of the catalytic cycle of Pol in real‐time.

## 2 Result and Discussion

**Figure 2:**
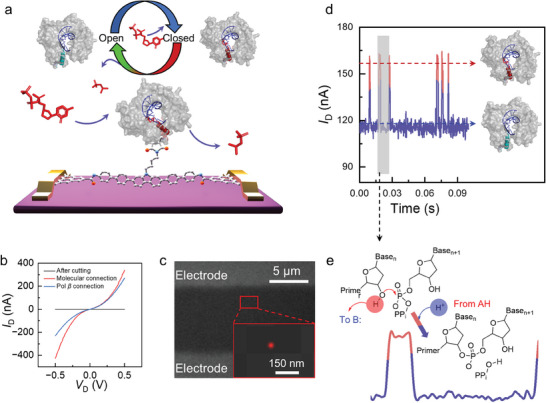
Single‐molecule electrical approach for real‐time monitoring the catalytic cycle of DNA synthesis catalyzed by human Pol *β* (hPol *β*). a) Schematic representation of a single‐molecule device for direct observation of nucleotide addition cycles catalyzed by a single DNA polymerase. b) Current against voltage (*I–V*) curves before and after preparation of the single‐molecule device. The red line shows the conductance after cutting; the black line shows the conductance after molecular connection; the blue line shows the conductance after further hPol connection. The voltage response indicates the successful preparation of the single‐molecule device. c) STORM image of a graphene‐molecule‐graphene single‐molecule junction with a single hPol attachment after treated by a 50 nm Cy3‐labeled DNA template. d) Current signal variations with a bias voltage of 200 mV between source and drain electrodes during DNA synthesis catalyzed by hPol at 37 °C. e) Reaction pathway of the nucleotide addition cycle catalyzed by hPol *β*.

To probe the catalytic mechanism of DNA synthesis catalyzed by hPol *β*, a single‐molecule platform was constructed by molecular engineering (**Figure** **2**a).^[^ ^23b^ ^]^ By introducing a thiol‐functional side arm through site‐specific mutation and the molecular bridge with a maleimide‐functionalized sidearm, a single Pol molecule was covalently assembled into the nanocircuit. The first step was to prepare graphene field‐effect transistors.^[^ ^24^ ^]^ Graphene was prepared on a copper sheet by chemical vapor deposition and then transferred from the copper sheet to silicon wafers. After photolithography by the template and thermal evaporation of metal electrode arrays, graphene field‐effect transistors were obtained (Figure S2, Supporting information). Next, graphene was exposed in the dash‐line window by electron‐beam lithography and etched by oxygen plasma, producing narrow gaps with carboxyl terminals.^[^ ^25^ ^]^ A molecular bridge with amino terminals and a maleimide functional center was covalently linked between a pair of graphene electrodes through amide bonds to construct stable GMG single‐molecule bridges.^[^ ^23b^ ^]^ Referring to the purification methods of Y265H hPol *β*,^[^ ^26^ ^]^ a mutant Pol molecule containing a sulfhydryl group near an *α*‐helix N was synthesized by W325C mutation and covalently linked to the molecular bridge by a thioether bond to construct stable GMG single‐molecule junctions (Figure S3, Supporting information). The results of the mass spectrometry characterization of the molecular bridge with a maleimide‐functionalized side arm are provided in Figure S4 (Supporting information). The activity tests of DNA Pol are provided in Figure S5 (Supporting information). The quantitative comparison of the enzyme activity before and after mutation is shown in Figure S6 (Supporting information). Moreover, based on the Michaelis–Menten equation, *k*_cat_ values for single nucleotide extension were calculated in Figure S7 (Supporting information). *k*_cat_ values of dATP, dCTP, dGTP, and dTTP incorporation catalyzed by WT and W325C Pol were summarized in Table S1 (Supporting information). Details of the protein mutation and device preparation are provided in the Supplementary Methods. The sequences of Pol before and after mutation are shown in Table S2 (Supporting information). Comparison of the current against voltage (*I–V*) curves before (no response) and after (some response) molecular integration confirmed the successful incorporation of the molecular bridge (Figure 2b; Figure S8, Supporting information). The *I–V* curve also showed some response after the protein was immobilized, which further proved the stability of the device (Figure 2b). The molecular junction based on a maleimide molecule is a *n*‐type field‐effect transistor. In the detection, Tris‐buffer (pH 7.8), hPol *β* is positively charged, which functions as an additional gate to reduce the concentration of charge carriers (electrons) and thus leads to the observed reduction in the device conductance.^[^ ^27^ ^]^ Also, *I–V* curves of six devices, with the same trend after protein attachment on the molecular bridge, are shown in Figure S9 (Supporting information), indicating the successful immobilization of protein. In order to visualize the immobilized hPol *β*, the Cy3 labeled DNA template was used to locate the position of protein.^[^ ^28^ ^]^ After cleaning with 20% Tween three times and with deionized water three times, the image of Stochastic Optical Reconstruction Microscopy (STORM) as shown in Figure 2c and Figure S10 (Supporting information) indicates a single fluorescence spot between the electrodes, proving the successful decoration of a single hPol *β*. After device fabrication, the structural change of Pol during catalyzing DNA synthesis was recorded by electrical signals in real‐time (Figure 2d). The reaction pathway of the nucleotide addition cycle catalyzed by Pol was observed step‐by‐step with conductance as the marker (Figure 2e).

Direct observation of the progressive nucleotide addition cycle catalyzed by Pols is a long‐term challenge. To avoid non‐synchronization, in this study, we used a single‐molecule approach^[^ ^23^, ^29^ ^]^ and focused on direct observation of the full catalytic cycle of hPol *β*. In addition, considering the excellent time resolution (time resolution in µs level) as well as high‐level molecular conductivity sensitivity toward conformational change and chemical reactions of GMG single‐molecule junctions,^[^ ^30^ ^]^ the Pol reaction mechanism can be monitored in real‐time by electrical signals. A bias voltage of 200‐mV is applied to the Pol‐modified molecular bridge at a temperature of 37 °C. The sampling nanoampere‐level current was sampled at 57.6 kHz. A current signal level, ≈120 nA, was detected. The detected current signal has no significant fluctuation, which mainly resulted from the instrument noise. We define this current signal level as the conductance state 1.

**Figure 3:**
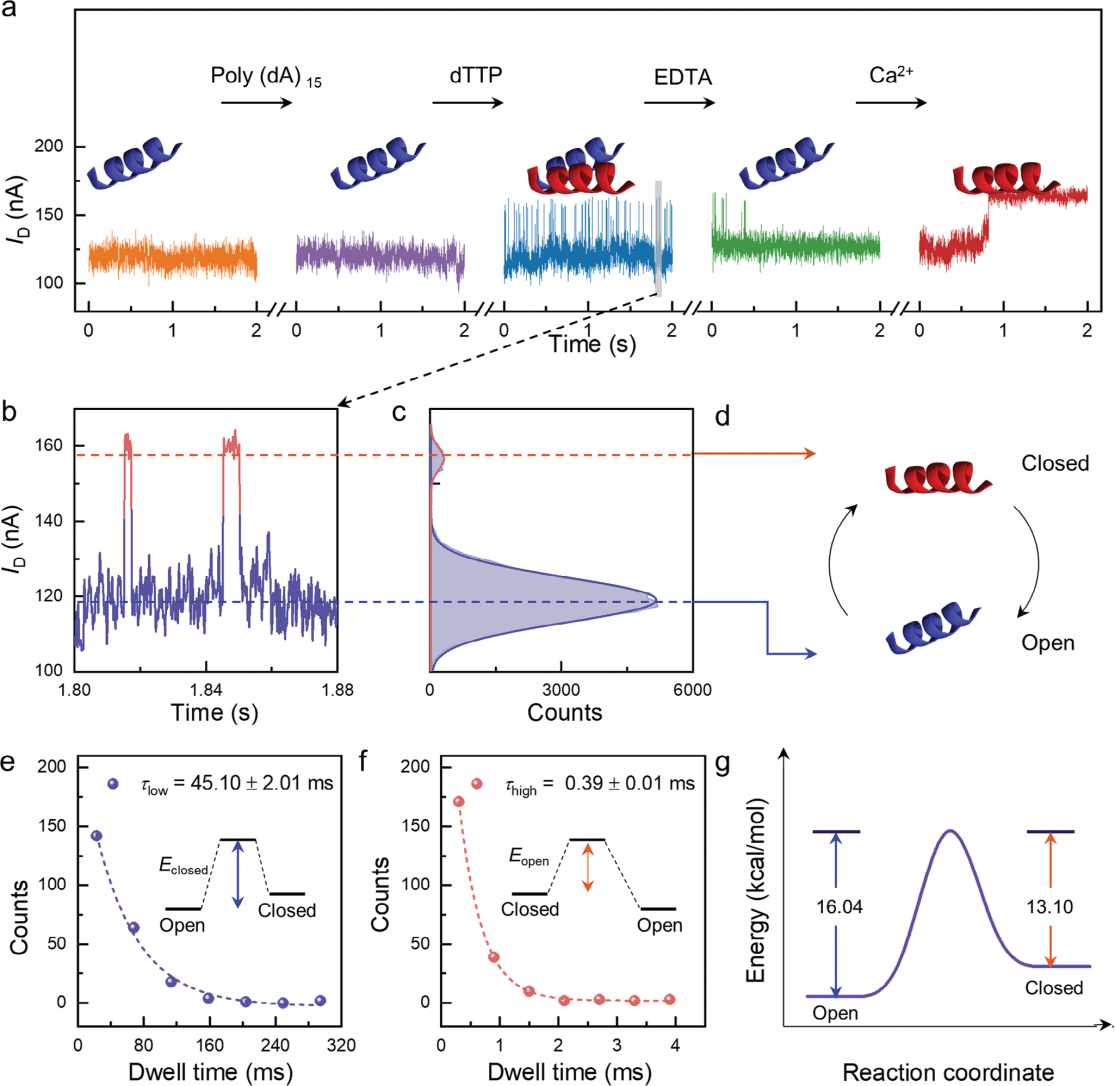
Electrical characterization and signal attribution of single‐molecule DNA synthesis catalyzed by human Pol *β* (hPol *β*). a) Current signal variations with a bias voltage of 200 mV between source and drain electrodes during the successive addition of the reaction substrates at 37 °C. The measurement was conducted first using the bare single hPol (orange), and then successively after injection of DNA template (purple), addition of dNTP (sky blue) and EDTA (green) as well as Ca^2+^ (red). b) Catalytic cycle (extracted from A) showing the two conductance states. c) Frequency distributions of all the current signals. d) Attribution of the two conductance states in the current signals from low to high, indicating the open and closed states of hPol *β*. e,f) Time interval plots of the (e) low‐ and (f) high‐conductance states derived from *I*–*t* trajectories. The distributions were fitted well by a single exponential decay function (dotted lines). The corresponding reaction pathways are also shown. g) Reaction coordinates of the catalytic cycle of hPol *β*. Activation energies were calculated using the Eyring equation ($E=RTln\frac{k_{B}T}{hk}$), where *R* = 8.314 Jmol^−1^•K^−1^, *k_B_* is the Boltzmann constant, and *h* is the Planck constant.

After adding a DNA template (Table S3, Supporting information) to the home‐made PDMS cube, as reaction cell covered on the Pol modified GMG single‐molecule junction, the current level remained at ≈120 nA, showing that the Pol structure did not change. With the addition of a dTTP solution, a new current signal level at ≈150 nA appeared, which is defined as conductance state 2. The two conducting states occurred alternately, which indicates a change in the Pol‐modified GMG single‐molecule junction structure (**Figure** **3**a). A Butterworth filtering method was used to reduce the circuit signal noise of the raw data (Figure S11, Supporting information). In combination with the template concentration optimization experiments at low concentrations, clustered electrical signals appeared, whose variation number is consistent with the number of newly incorporated bases, demonstrating that DNA polymerase is inserting for each current signal variation (Figure S12, Supporting information). Another set of data in Figure S13 (Supporting information) also testified that a new conductance state, with higher current level, appeared and two conducting states occurred alternately after injection of dNTP solution. Therefore, this change in current signal we detected was initially assumed to arise from the open and closed states of Pol during the nucleotide addition cycle. To ensure that the observed conductance changes are due to the polymerase activity and not due to the device instability or environmental factors, the device was monitored continuously for 60 s without the introduction of DNA or dNTPs (Figure S14, Supporting information). No obvious fluctuations in the *I–t* curves and the single peak in the Gaussian fitting indicate the reliability of our devices. Under the same conditions, systematic control experiments demonstrated that there were no obvious fluctuations in the currents for the devices before connection (open circuit) (Figure S15, Supporting information) and after connecting the molecular bridge (Figure S16, Supporting information), and graphene nanoribbon devices (Figure S17, Supporting information). These results demonstrate that the current change raised from Pol during DNA polymerization and the potential noises were excluded. To confirm that the electrical conductance fluctuations result from conformational changes in hPol *β*, the device was characterized by an alternative method using STORM. In the experiment of simultaneously recorded fluorescent and electrical signals under addition of DNA template and cy3‐labeled dNTP (γ‐[(6‐Aminohexyl)‐imido]‐dATP‐Cy3), synchronization in the fluctuation of the bistable electrical and fluorescent signals clarified that the alternation in conductance originated from structural changes on the active site of DNA polymerase initiated by dNTP (Figure S18, Supporting information). Previous studies proved that EDTA coordinated divalent ions in solution and then kept the polymerase in the open state, and that Ca^2+^ did not support catalysis and trapped DNA Pol in the closed state.^[^ ^5b^ ^]^ After the addition of an EDTA solution, the current dropped to ≈120 nA (conductance state 1), which implies an open state. The addition of Ca^2+^ significantly increased the current to ≈150 nA (conductance state 2), which implies a closed state (Figure 3a). Therefore, after the addition of dTTP, sequential periodic transitions of the current levels (Figure 3b) between the two stable states as shown in the *I*–*t* curve (conductance states 1 and 2, and the frequency statistics in Figure 3c) correspond to the open and closed states of Pol (Figure 3d). This finding shows that the open and closed states of Pol can be monitored in real time and that it is feasible to obtain the dynamical behavior of the two species separately. The mean duration of the open complex ⟨*τ*_low_⟩ was ≈45.1 ms and that of the closed complex ⟨*τ*_high_⟩ was ≈0.39 ms for the homopolymeric DNA templates (Figure 3e,f). We calculated activation energies using the Eyring equation as ≈16.04 Kcal mol^−1^ for the closed conformational transition and ≈13.10 Kcal mol^−1^ for the open conformational transition (Figure 3g). Based on the detection on our constructed GMG single‐molecule detection platform, the stable state is enzyme's open conformation, including all steps except catalysis, such as the non‐covalent binding step of the dNTPs and the release of mismatched dNTPs. Therefore, the rate‐limiting step is the non‐covalent binding step occurring during the enzyme's open conformation.

**Figure 4:**
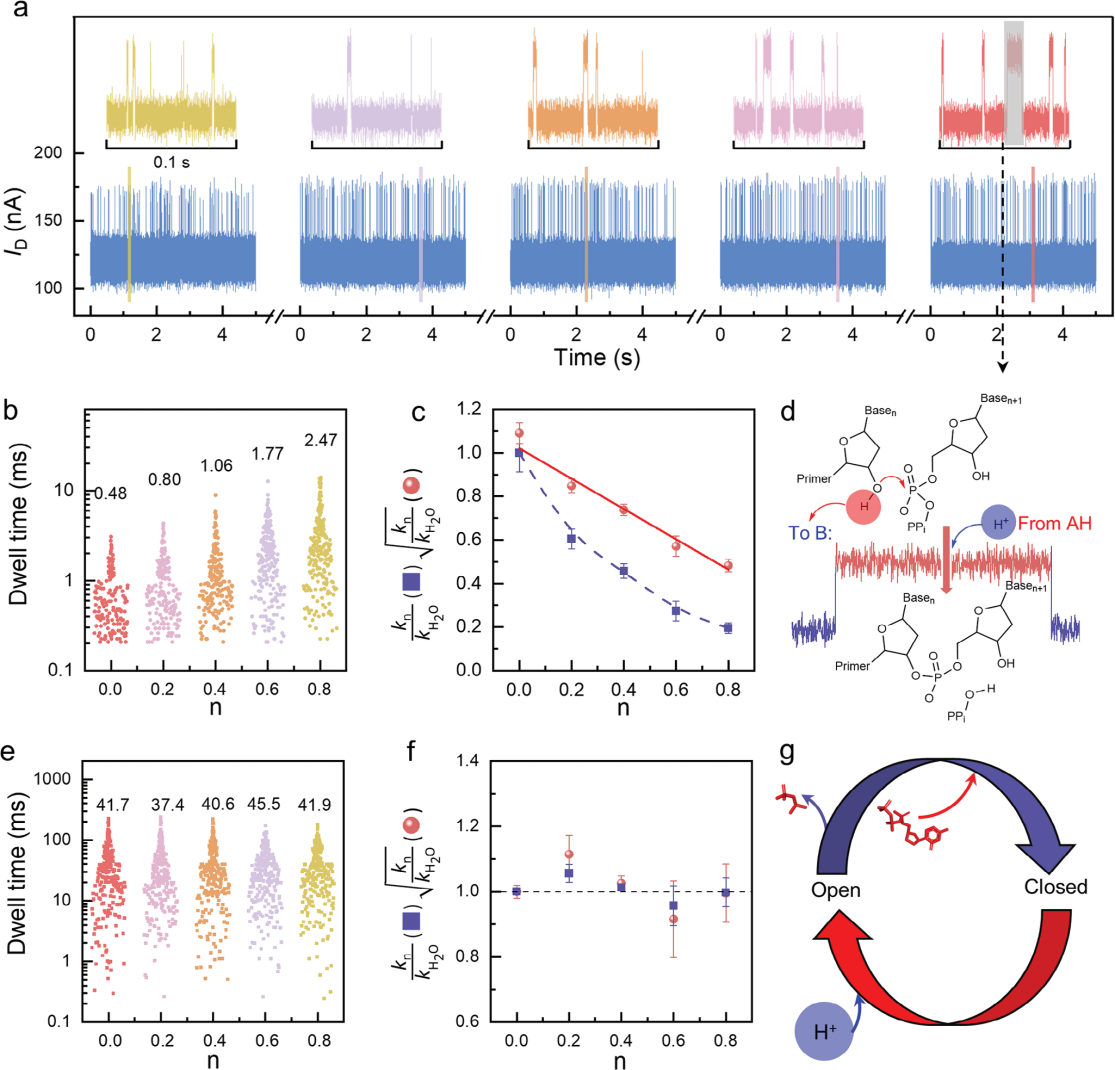
Dynamic analyses of the catalytic cycle of human Pol *β* (hPol *β*). a) Nucleotide incorporation assay. *I*–*t* curves (10 s) in five‐mole fractions of D_2_O (0, 0.2, 0.4, 0.6, and 0.8) at 200 mV (bottom) and selected time windows with lengths of 0.1 s (top). b) Mean duration of the open state. c) Mean duration of the closed state. d) Protons transfer in a nucleotidyl transfer reaction occur in closed state. e) Two protons are transferred during nucleotidyl transfer. *k_n_* is the observed rate constant for nucleotide incorporation at a particular mole fraction of D_2_O, $k_{H_{2}O}$ is the observed rate constant for nucleotide incorporation in H_2_O, and n is the mole fraction of D_2_O. Values for $k_{n}/k_{H_{2}O}$ (filled squares) or the square root of $k_{n}/k_{H_{2}O}$ (filled circles) are plotted as a function of n. f) Proton transfer did not occur during the rate‐limiting step in the open state. The dotted line indicates that the fit of the data is close to a line. g) PP_i_ formation occurs before the reverse conformational change.

**Figure 5:**
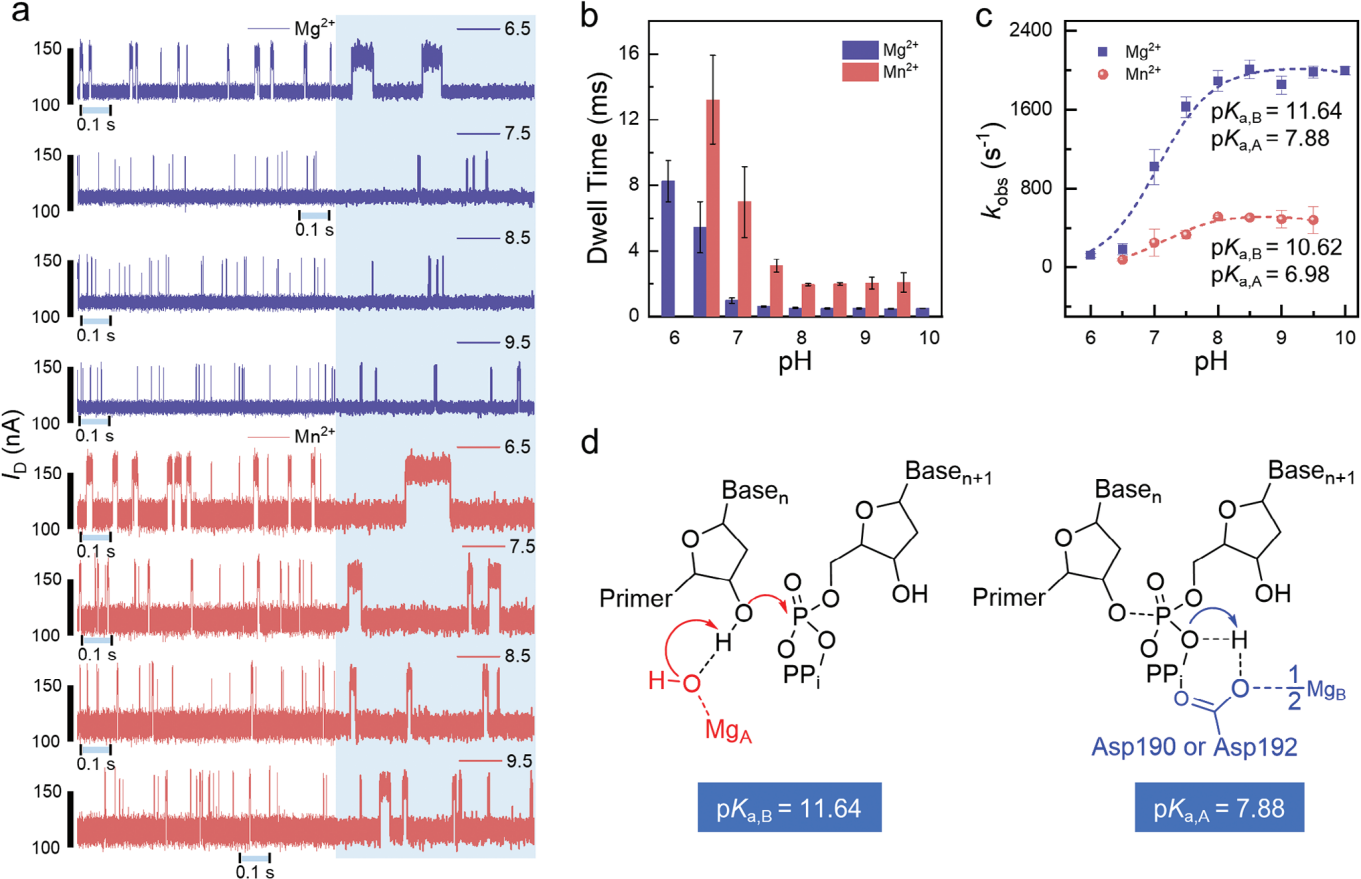
Mechanism of the catalytic cycle of human Pol *β* (hPol *β*). a) pH dependence of the nucleotidyl transfer reaction. *I*–*t* curves (1 s) at four pHs (6.5, 7.5, 8.5, and 9.5). Electrical trajectories marked in blue below the curves show the 0.1 s magnified view of each trace. Complete *I*–*t* curves (10 s) at nine pHs (6.0–10.0) for Mg^2+^ and at seven pHs (6.0–10.0) for Mn^2+^ are provided in Figures S24 and S25 (Supporting Information). b) Mean duration of the open state at different pH values. c) The obtained p*K*_a_ values of ≈11.64 for Mg^2+^ (filled squares) and ≈10.62 for Mn^2+^ (filled circles). The p*K*_a_ values were calculated by fitting to the equation $k_{obs}=\frac{k_{max}}{1+10^{pk_{a,A-pH}}+10^{pH_{-}pk_{a,B}}}$. d) Obtained reaction pathways for Mg_A_ and Mg_B_. Solvated metal hydroxide as a general base deprotonates the 3′‐OH nucleophiles. D190 or D192 acts as a potential proton donor to PP_i_.

If proton transfer is the rate‐limiting step in a nucleotidyl transfer reaction, the isotope effect will be apparent.^[^ ^31^ ^]^ To test the rate‐limiting step, a solvent deuterium isotope experiment was conducted. *I*–*t* curves (**Figure** **4**a and the complete 10s *I*–*t* curves in Figure S19 (Supporting information) were recorded at different mole fractions of D_2_O (0, 0.2, 0.4, 0.6, and 0.8). The *τ*_high_ increased successively, indicating slower reaction rates at higher concentrations of D_2_O (Figure S20, Supporting information). However, with the increase of D_2_O concentration, the *τ*_low_ remains almost constant (Figure S21, Supporting information). The *I*–*t* curves were recorded on more than one device (another set of data are provided in Figure S22, Supporting information). Similarly, the *τ*_high_ increased with the increase of D_2_O concentrations (Figure S23, Supporting information), and the *τ*_low_ were not affected by the increased concentration of D_2_O (Figure S24, Supporting information). Therefore, a significant isotopic effect ($k_{n}/k_{H_{2}O}$) was observed in the high state, where *k*_n_ is the observed rate constant for the nucleotide incorporation reaction measured in different mole fractions *n* of D_2_O, and $k_{H_{2}O}$ is the observed rate constant for the reaction in H_2_O. The real‐time monitoring of this single‐molecule method allowed the spatial and temporal properties of the open and closed complexes to be detected. The mean duration of the open state increased when the *n* of D_2_O increased (Figure 4b), but no significant change was detected in the closed state (Figure 4c). The results indicate that protons transfer in a nucleotidyl transfer reaction occur in closed state (Figure 4d). Moreover, the number of protons transferred was quantified by proton inventory studies based on the modified Gross‐Butler equation ($k_{n}/k_{H_{2}O}=\left(1-n+n\phi_{1}\right)\;\left(1-n+n\phi_{2}\right)\left(1-n+n\phi_{3}\right)\text{…}$). If only one‐proton transfer occurs at the rate‐limiting step, it conforms to the single‐proton transfer model, where $k_{n}/k_{H_{2}O}=\left(1-n+n\phi_{1}\right)$ and the quotient of $k_{n}/k_{H_{2}O}$ is linear with *n*. If two‐proton transfer occurs at the rate‐ limiting step, it conforms to the double‐proton transfer model, where $k_{n}/k_{H_{2}O}=\left(1-n+n\phi_{1}\right)\;\left(1-n+n\phi_{2}\right)$ and the square root of the quotient of $k_{n}/k_{H_{2}O}$ is linear with *n*. For the data of the open state, the ration of $k_{n}/k_{H_{2}O}$ not linear with *n*, indicating that there is not a single‐proton transfer process. In contrast, the square root of the quotient of $k_{n}/k_{H_{2}O}$ as a function of *n* is linear (Figure 4e; Figure S25, Supporting information), which fits well to the double‐proton transfer model, suggesting that two‐proton transfer occurred at the open state. For the data of the closed state (Figure 4f), the quotient of $k_{n}/k_{H_{2}O}$ fluctuated ≈1, indicating that no significant isotopic effect was detected. On the basis of these data, we concluded that two‐proton transfer reactions occurred in the rate‐limiting transition state for phosphodiester bond formation catalyzed by hPol *β*, suggesting that both deprotonation of the 3′‐OH nucleophile and protonation of the PP_i_ leaving group occurred in the transition state of phosphodiester bond formation. Interpretation of these data in the context of known Pol structures suggests the presence of a general base for deprotonation of the 3′‐OH nucleophile, although the use of a water molecule and a general acid for protonation of the PP_i_ leaving group cannot be conclusively ruled out. Moreover, two‐proton transfer occurred in a closed state, directly indicating that the PP_i_ formation occurred before the reverse conformational change (Figure 4g). Recently, time‐resolved crystallographic studies of Y‐ and X‐family DNA polymerases, including human DNA hPol *β*, have provided substantial evidence to promote an expansion of the two‐metal ion mechanism to the third divalent metal ion mechanism, the precise role of which is currently debated.^[^ ^4c^ ^]^ Significantly, the additional Mg^2+^ is not observed in the structures that have a substantial population of reactant species. Considering that it is observed only in product complexes, the additional Mg^2+^ usually defined as the PP_i_‐leaving metal.^[^ ^6a^ ^]^ Based on the theory of the isotope effect and solvent deuterium isotope experiments, we have proposed a two‐proton transfer mechanism in the DNA replication reaction catalyzed by hPol *β*, which confirms that the additional Mg^2+^acts as a PP_i_‐leaving metal. To probe the catalytic mechanism of the catalytic cycle of hPol *β*, we examined the pH dependence for the nucleotide addition cycle in Mg^2+^ and Mn^2+^ complexes (**Figure** **5**a) and *I*–*t* curves (10 s) at nine pHs (6.0–10.0) in Mg^2+^ and at seven pHs (6.0–10.0) in Mn^2+^ (Figures S26 and S27, Supporting information). The *τ*_high_ values were statistic in Figure S28 (Supporting information) for Mg^2+^ and in Figure S29 (Supporting information) for Mn^2+^.

The results using another set of data are provided in Figures S30 and S31 (Supporting information). Moreover, Figure S32 (Supporting information) shows the statistics of *τ*_high_ in Mg^2+^ and Figure S33 (Supporting information) shows the statistics of *τ*_high_ in Mn^2+^. The effect of pH on the mean duration of the open state is shown in Figure 5b. The observed rate constant increased at pH 6.5–7.5 with a slope of ≈1 (Figure 5c). The observed rate constant was pH insensitive at pH 7.5–8.5 and reduced slightly at pH 8.5–9.5. Consistent with the nucleotide incorporation assay, in both Mg^2+^ and Mn^2+^, a bell‐shaped curve was observed, indicative of two ionizable groups. This finding further supports two‐proton transfer, implying that deprotonation of 3′‐OH of the primer and protonation of the PP_i_ leaving group occurred before the reverse conformational change.

To identify a general base for deprotonation of the 3′‐OH nucleophiles and a general acid for protonation of the PP_i_ leaving group, we examined the role of metal ions in the nucleotidyl transfer reaction. Mg_A_ is believed to lower the p*K*_a_ of the primer 3′‐OH, and a deprotonated Mg_A_‐bound OH^−^ (p*K*_a_ ≈11.40) is a candidate for the general base because the p*K*_a_ of these species was >9 (Figure S34, Supporting information). The reaction was conducted using 10 mM of two metals (Mg^2+^ and Mn^2+^ of similar size) with different p*K*_a_. The unperturbed p*K*_a_ of a water molecule coordinated to Mg^2+^ or Mn^2+^ was ≈11.40 and 10.60, respectively.^[^ ^32^ ^]^ Moreover, the Mg_A_‐bound OH^−^ was located near the 3′‐OH nucleophile sites,^[^ ^14^ ^]^ and therefore could act as a general base and either directly or indirectly deprotonate the 3′‐OH nucleophiles. The binding rate of the nucleotidyl transfer reaction was calculated at different pH values in Mg^2+^ or Mn^2+^. The p*K*_a_ of the general base was ≈11.64 for Mg^2+^ and ≈10.62 for Mn^2+^, which is consistent with the p*K*_a_ of Mg_A_‐bound OH^−^ (Figure 5c; Figure S35, Supporting information). This finding directly proved that Mg_A_‐bound OH^−^ acted as the general base for proton transfer.

Moreover, the p*K*_a_ of the general acid was ≈7.88 for Mg^2+^ and ≈6.98 for Mn^2+^. In the crystal structure of hPol *β*, D190 and D192 are near the PP_i_ (PDB ID: 4KLG). Alberts et al.^[^ ^8c^ ^]^ proposed that D190 acts as a potential proton donor to PP_i_ based on mixed QM/MM calculations. In hPol *β*, D190 and D192 have the potential for protonation, but the p*K*_a_ for the free carboxyl group is relatively low at 3.65. Perturbation of the p*K*_a_ of acid groups to near neutrality has been observed under metal ion coordination conditions^[^ ^33^ ^]^ (The mechanism of COO^−^−1/2Mg^2+^ formation is shown in Section S8, Supporting information), which suggests that D190 or D192 coordinated with Mg_B_ may serve as the general acid catalysis in hPol *β*. Moreover, a △p*K*_a_ of 0.90 was observed under Mg^2+^ and Mn^2+^ coordination conditions. A p*K*_a_ shift of 0.9 is consistent with the △p*K*_a_ of 0.80 for Mg^2+^ and Mn^2+^ (the difference between 11.40 and 10.60 for Mg^2+^ and Mn^2+^, respectively), which suggests that the free carboxyl group of D190 or D192 coordinated with Mg_B_ may participate in proton transfer. This directly demonstrates that the third Mg^2+^ serves as a PP_i_‐leaving metal, with its primary role being to assist in the release of PP_i_. Therefore, our experimental results have clarified the role of the third Mg^2+^ in the catalytic mechanism of hPol *β*. Consequently, we proposed a framework for the mechanism of the catalytic cycle of hPol *β*, where Mg_A_‐bound OH^−^ acts as the general base for proton transfer, and D190 or D192 coordinated with Mg_B_ acts as a proton donor to PP_i_ (Figure 5d). Details of the catalytic mechanism of Pol are provided in Figure S36 (Supporting information).

Moreover, considering our method of constructing the platform for protein fixation through protein mutation, we believe that the GMG single‐molecule junction platform for DNA for hPol *β* catalysis mechanism is widely applicable to the study of other enzyme catalytic mechanisms, especially DNA and RNA polymerases mechanisms. For example, by utilizing this platform, the characterization of nucleotide‐binding in mismatched substrates, which typically does not display isotope effects, has been achieved (Figure S37, Supporting information).

## 3 Conclusion

The use of a single Pol molecule as a real‐time observation object for direct observation of progressive Pols enabled us to provide additional evidence to important issues in the reaction mechanism: i) the order between the release of PP_i_ and the reverse conformational change; and ii) the proton transfer pathway of the 3′‐OH nucleophile nucleophiles and PP_i_. The solvent deuterium isotope experiment indicated that the rate‐limiting step of the PP_i_ formation was proton transfer, which occurred before a reverse conformational change. By detection of the p*K*_a_, Mg_A_‐bound OH^−^ (p*K*_a_ ≈ 11.64) was shown to act as a general base, transferring the proton and activating the nucleophile of 3′‐OH. D190 or D192 coordinated with Mg_B_ served as a general acid that catalyzed the departure of the PP_i_ group. Clarification of the DNA polymerase catalytic reaction mechanism provides crucial insights into further designing high‐performance Pols, tracing the origin of replication and translation errors, and building a single Pol‐based real‐time DNA sequencing platform. In addition to offering a complete understanding of the detailed mechanism of Pols catalysis, this work highlights the potential of the single‐molecule technique for visualizing the intrinsic dynamic process of broad biomolecular interactions at single‐molecule resolution.

## Conflict of Interest

The authors declare no conflict of interest.

## Supporting information



Supporting Information

## Data Availability

The data that support the findings of this study are available from the corresponding author upon reasonable request.
