# Supplementary material for: Unveiling the Mechanism of Deprotonation and Proton Transfer of DNA Polymerase Catalysis via Single‐Molecule Conductance
Source: Adv Sci (Weinh). 2024 Nov 21;12(2):2408112. doi: 10.1002/advs.202408112 (PMC11727276; doi:10.1002/advs.202408112)
Supplement: Supplementary file 1 — Supporting Information [file ADVS-12-2408112-s001.pdf]

ADVANCED SCIENCE
Open Access

Supporting Information

for *Adv. Sci.*, DOI 10.1002/advs.202408112

Unveiling the Mechanism of Deprotonation and Proton Transfer of DNA Polymerase Catalysis via Single-Molecule Conductance

Lihua Zhao, Yang Xu, Zhiheng Yang, Wenzhe Liu, Shichao Zhong, Jingwei Bai and Xuefeng Guo**

Supporting Information

Unveiling the mechanism of deprotonation and proton transfer of DNA polymerase catalysis via single-molecule conductance

Lihua Zhao†[a], Yang Xu†[b], Zhiheng Yang[a], Wenzhe Liu[a], Shichao Zhong[a], Jingwei Bai*[b], and Xuefeng Guo*[a,c]

[a] L. Zhao, Dr. Z. Yang, Dr. W. Liu, S. Zhong, Prof. X. Guo
Beijing National Laboratory for Molecular Sciences, National Biomedical Imaging Center
College of Chemistry and Molecular Engineering, Peking University
292 Chengfu Road, Haidian District, Beijing 100871, P. R. China
Email: guoxf@pku.edu.cn

[c] Y. Xu, Prof. J. Bai
School of Pharmaceutical Sciences, Tsinghua University
Beijing, 100093, P. R. China
Email: jingwbai@mail.tsinghua.edu.cn

[d] Prof. X. Guo
Center of Single-Molecule Sciences, Institute of Modern Optics, Frontiers Science Center for New Organic Matter
College of Electronic Information and Optical Engineering, Nankai University
38 Tongyan Road, Jinnan District, Tianjin 300350, P. R. China

[†] These authors contributed equally to this work.

Supporting information for this article is given via a link at the end of the document.

Table of Contents

Section 1: Materials and Methods

Section 2. Expression and purification of hPol *β*

Section 3: The catalytic cycle and structure of hPol *β*.

Section 4. Device Fabrication and Molecular Connection

Section 5. hPol *β* activity test

Section 6. Electrical Characterization and Control Experiments

Section 7. Theoretical Calculations

Section 8. The equation for the formation of COO^{-}-1/2Mg^{2+}

Section 9. Sequence of the DNA hPol *β* and DNA template

References

Section 1: Materials and Methods

Real-time electrical measurements. A home-made PDMS cube, contained a hole ~2 mm in diameter, as a reaction chamber, was covered on the GMG single-molecule junction after modified with Pol.[1] In a standard buffered solution (10 mM Tris, 50 mM NaCl, 10 mM $MgCl_2$, 10 mM TCEP, pH 7.8), the device was measured with and without the template containing a standard M13F priming site (poly(dA)$_{15}$). Then, the reaction hybridization protocol involved heating template (50 nM) and M13F (50 nM) in buffer to 90 °C for 1 min and slowly cooling to 25 °C. After hybridization with the M13F forward primer, either complementary, the template was injected into the microchamber. Then, dTTP (10 μM), the substrate of the enzyme, was added. To prove the correspondence between the conductive states and the structures of hPol β, buffers (10 mM Tris, 50 mM NaCl, 10 mM $CaCl_2$, 10 mM TCEP, pH 7.8 and 10-mM Tris, 50 mM NaCl, 10 mM EDTA, 10 mM TCEP, pH 7.8) were prepared. Then, by a home-made PDMS microfluidic device, the template and dNTP dissolved in a specific buffer containing Mg^{2+}, EDTA, and Ca^{2+} were injected slowly in turn. During the test, the chamber temperature was controlled by INSTEC hot/cold chuck. It involves a proportion-integration-differentiation control system (± 0.001 °C) and a liquid nitrogen cooling system. Using an HF2LI Lock-in Amplifier (Zurich Instruments), the source-drain voltage was kept at DC 200 mV throughout all real–time electrical measurements.

Solvent deuterium kinetic isotope effect and proton-inventory experiments. The solvent deuterium kinetic isotope effect and proton-inventory experiments were performed by directing observation of the DNA synthesis process catalyzed by Pols. We prepared hPol β, templates, dNTP and buffers in 100 % H_2O or 100 % D_2O. Then, mixed them in specific proportions to obtain different proportions of D_2O (0 %, 20 %, 40 %, 60 % or 80 %). For the D_2O solutions, the pD was used to instead of pH and was adjusted according to pD = pH + 0.4. The values of the solvent deuterium kinetic isotope effect were calculated as the ratio of k_{pol} values obtained in H_2O divided by that obtained in D_2O ($\frac{k_n}{k_{H_2O}}$).[2]

pH dependent experiment for nucleotidyl-transfer reaction. The buffers used to maintain the desired pH were Mes (pH 5.7–6.5), Tris (pH 7.0–9.2), and Ches (pH 9.2–10.0). To accommodate the transition metal ions, Bis-Tris [2,2-bis(hydroxymethyl)-2,2',2''-nitrilotriethanol], equal in concentration to the metal, was added. Buffers of different pHs between 6.0 and 10.0 in 10 mM Mg^{2+}: 6.0, 6.5, 7.0, 7.5, 8.0, 8.5, 9.0, 9.5 and 10.0, and pHs between 6.5 and 9.5 in 10 mM Mn^{2+}: 6.5, 7.0, 7.5, 8.0, 8.5, 9.0, and 9.5 were prepared. We prepared hPol β, templates and dNTP in the above different pH buffers. Moreover, during the test, to avoid cross-contamination of different pH buffers, the nanocircuit was rinsed extensively with new buffer before the addition of each new buffer.

Statistical analysis. To better analyze the current data, we used the Butterworth filtering method to perform low-pass filtering to reduce the circuit signal noise of the raw data. 0.5 kHz was the stopband frequency, and the other, 5 kHz, was the start of the stopband attenuation. The sequent data processing was carried out by MATLAB 2016b. A QuB software was used to idealize the filtered data for obtaining the dwell time of each signal event and the number of total events based on the hidden Markov model. Origin 2019b was finally utilized to analyze extracted data. Relying on the exponential decay function, the statistical average dwell time was obtained. All statistical data are presented as mean ± SD.

Section 2: Expression and purification of human Pol *β* (hPol *β*).

The human Pol *β* (hPol *β*) W325C mutant gene fused with 6×His-tag at the N-terminal was subcloned into pJE401, which transformed in BL21(DE3) competent cells.[3] Monoclonal colony was inoculated into LB with Kanamycin and then protein was induced overnight with IPTG when bacteria grown to OD_{600} = 0.6~0.8. Cells were centrifugated to harvest and lysed with homogenizer. Protein in supernatant was precipitated by saturated ammonium sulfate overnight at 4 ℃ and loaded onto a column with Ni^{2+} resin. Target protein was eluted from Ni^{2+} resin with 300 mM imidazole after washing with 20 mM imidazole. The elution was purified with heparin column on AKTA and eluted with NaCl gradient. Fractions were collected and the target protein was analyzed by SDS-PAGE. The target fraction was then concentrated and further purified with SEC column. 50 % glycerol was used for protein storage at –20℃.

Section 3: The catalytic cycle and structure of hPol *β*.

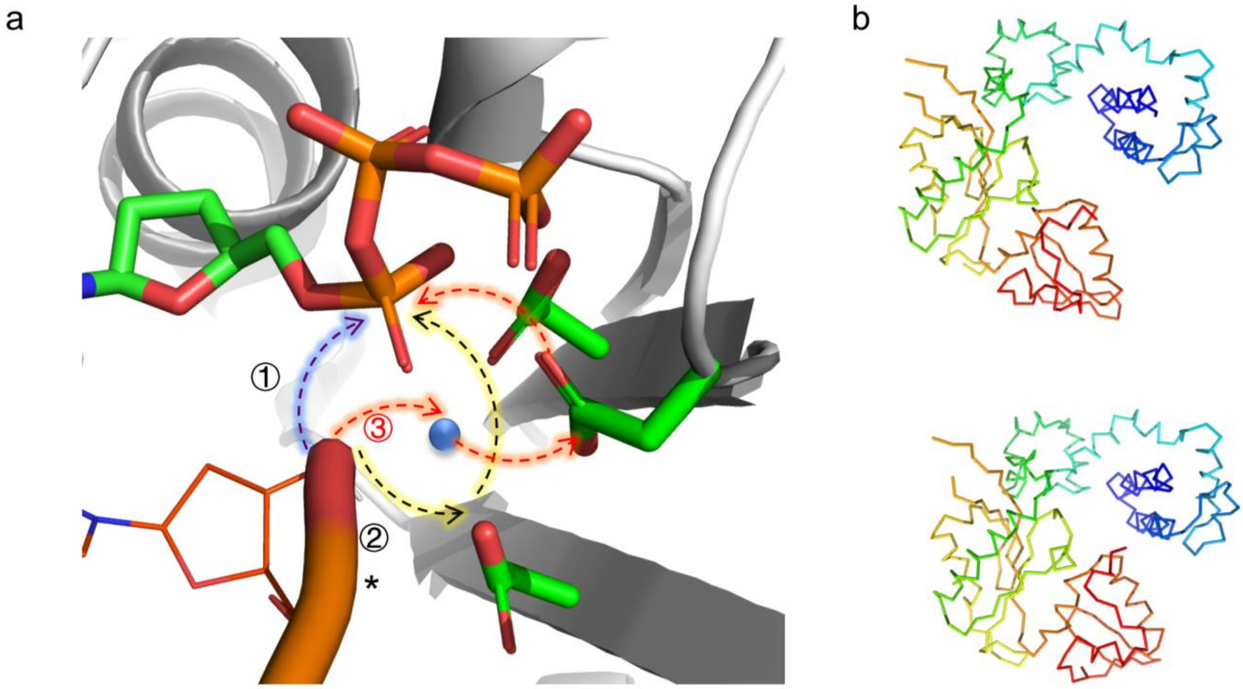

Figure S1. The catalytic cycle and structure of hPol *β*. (a) Possible sketch of reaction pathways: ① Arrows show direct deprotonation of the terminal primer 3'-OH to $O_{2\alpha}$ of the incoming dNTP; ② Arrows show direct deprotonation of the terminal primer 3'-OH to the active site Asp residues; ③ Arrows show proton–transfer steps from the terminal primer 3'-OH to pyrophosphate, via active H_2O and D190.[4] (b) The image depicts open (above) and closed (below) structures of a homologous DNA polymerase (hPol *β*, PDB: 2BPG and 1BPZ).

Section 4. Device Fabrication and Molecular Connection

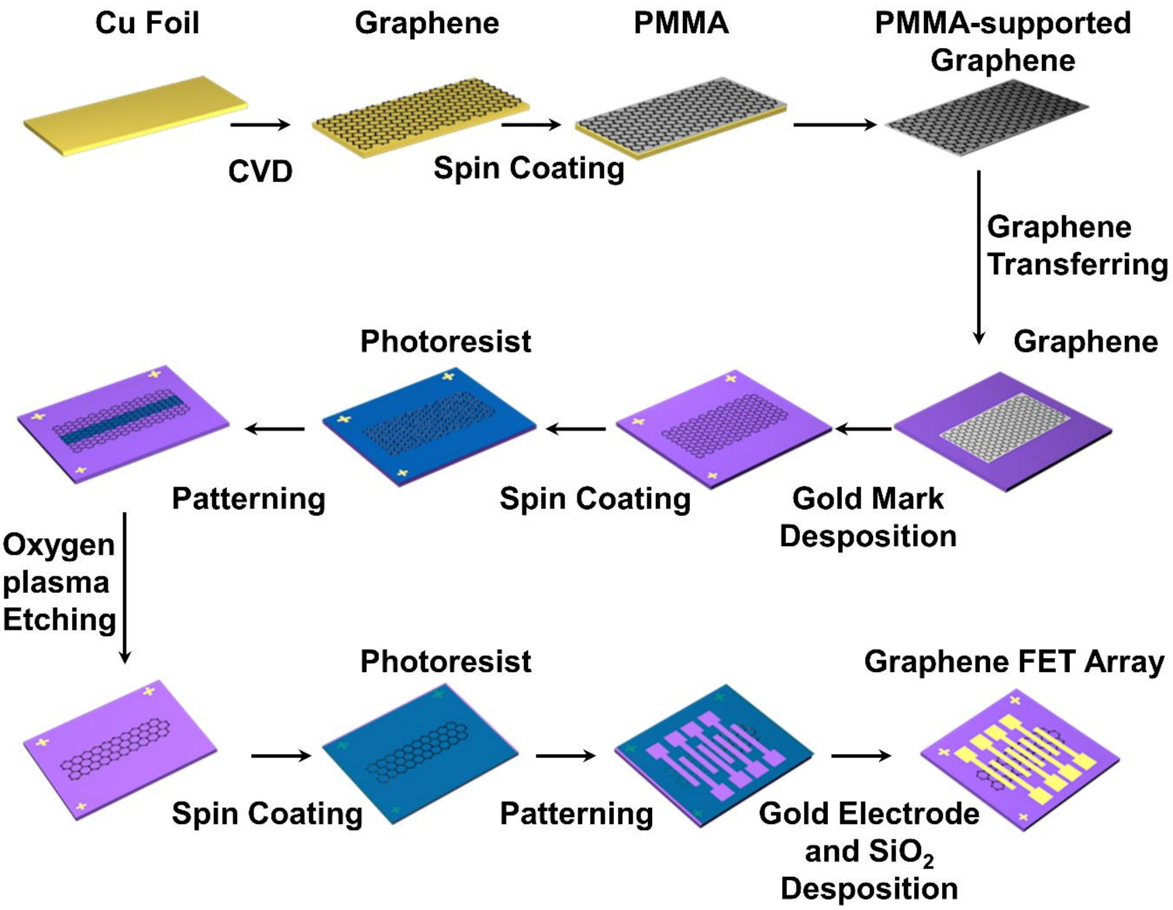

Figure S2. Fabrication of graphene field-effect transistors (FETs).

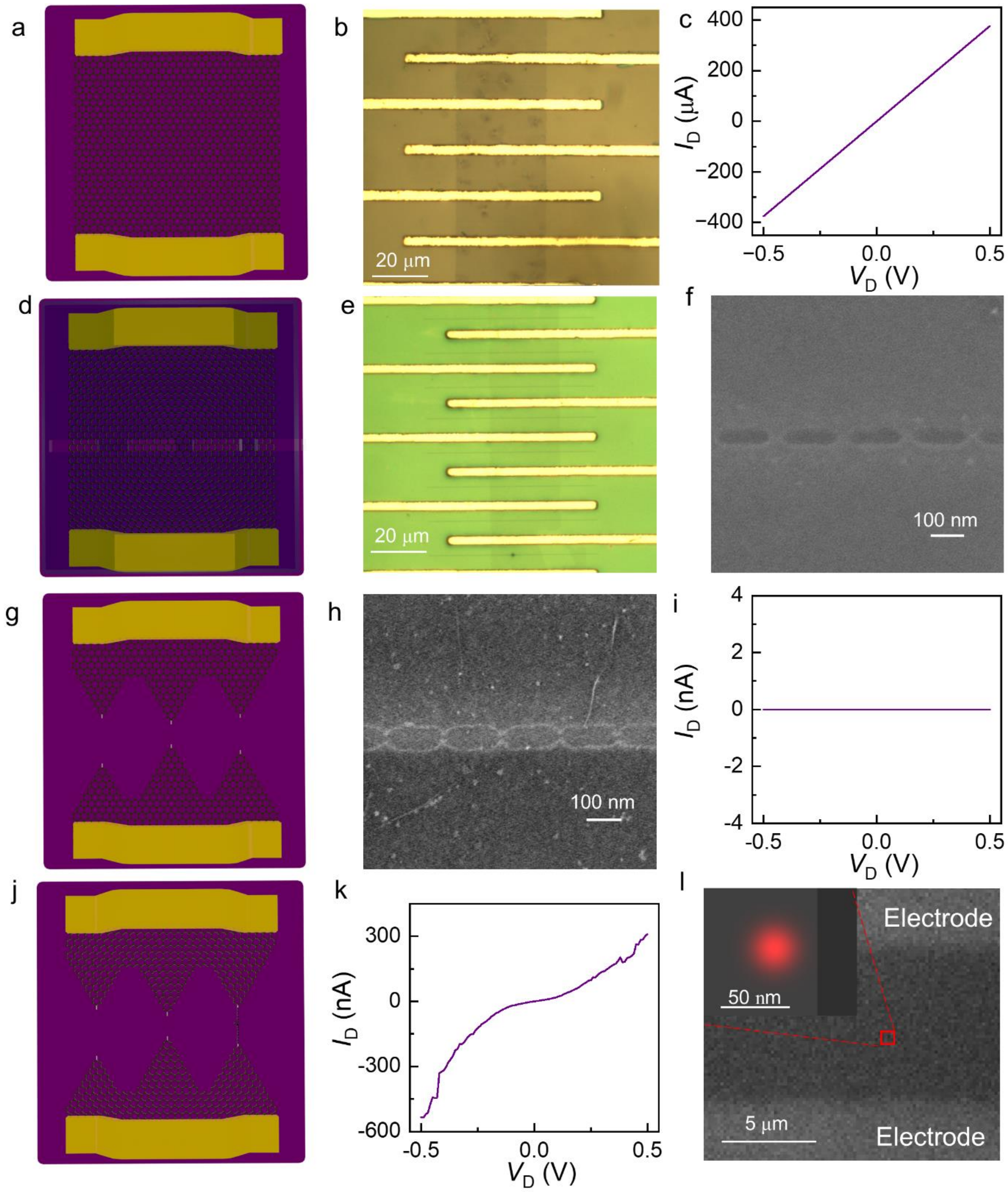

Figure S3. Schematic of a procedure of single-molecule device fabrication. (a) Schematic of the initial stage of graphene FETs. (b) Corresponding photograph of the initial stage of graphene FETs. (c) *I*−*V* scan between one pair of gold electrodes. (d) After electron-beam lithography (EBL). (e) Photograph of the initial stage of graphene FETs after EBL. (f) Corresponding SEM photograph of graphene FETs after EBL. (g) Graphene electrode array after oxygen plasma etching. (h) Corresponding SEM photograph of a graphene electrode array. (i) *I*−*V* scan between one pair of open circuit with graphene point contacts. (j) After molecular connection. (k) *I*−*V* scan between one pair of gold electrodes after molecular connection. (l) Super-high-resolution image of the single-protein site obtained by stochastic optical reconstruction microscopy (STORM).

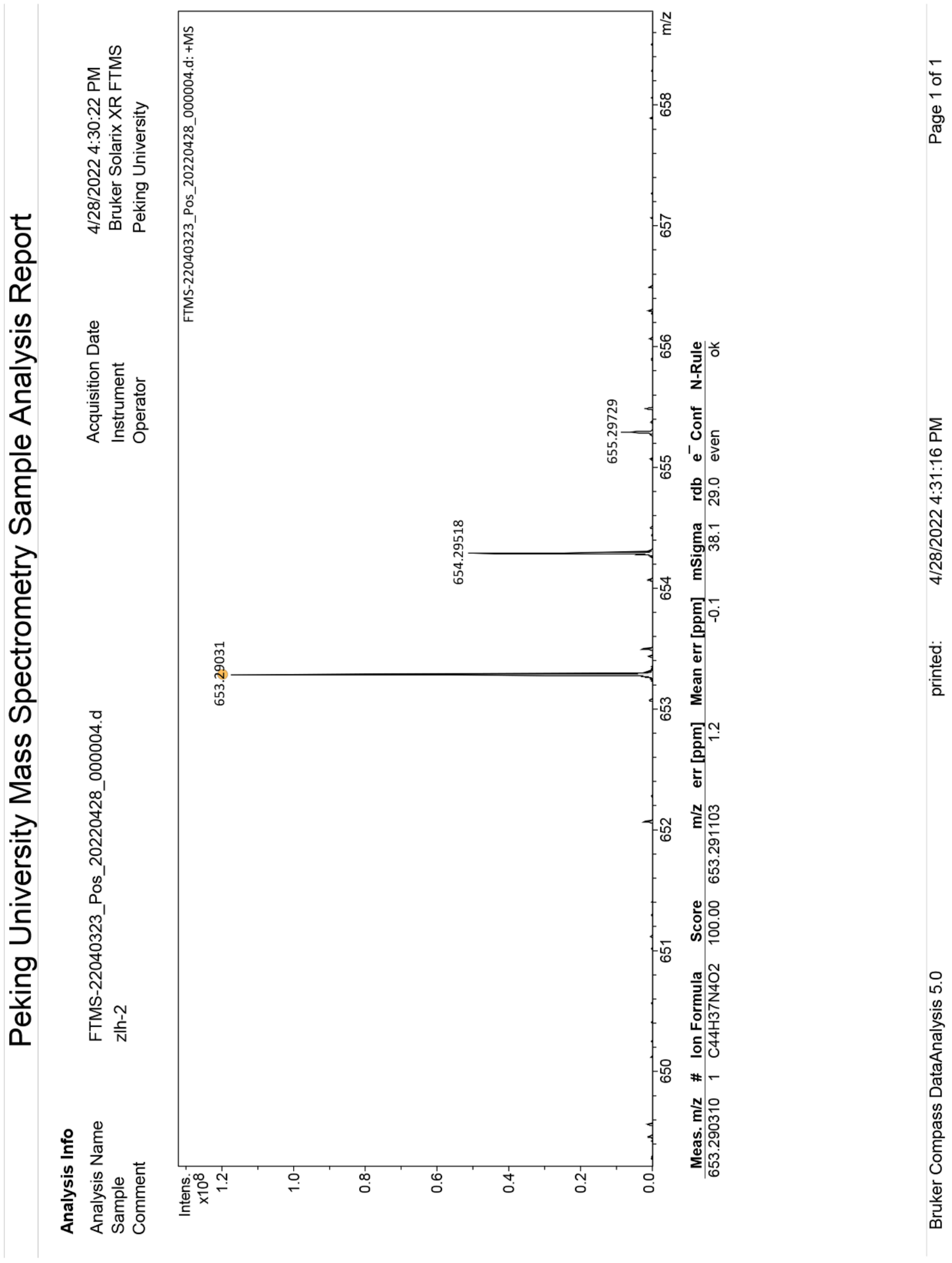

Figure S4. HRMS spectrum of the molecule bridge with amino terminals and a maleimide-functionalized side arm.

Section 5. DNA polymerase activity test

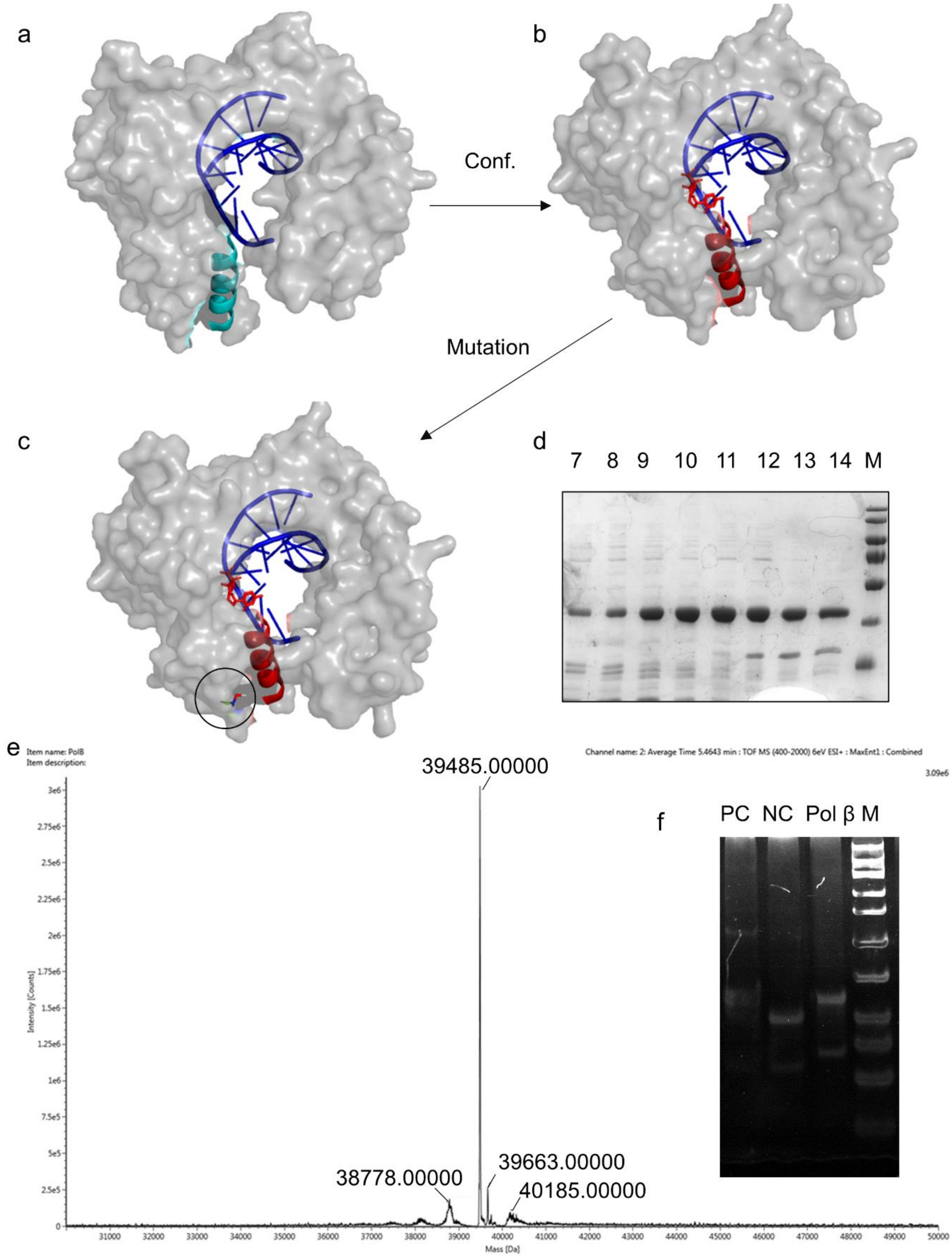

Figure S5. Mutation site and activity tests of DNA hPol *β*. (a) Crystal structure of hPol *β* after binding DNA template chain (PDB ID 4KLG). The O helix is marked with blue. (b) Crystal structure of hPol β after binding dNTP (PDB ID 4KLU). A conformational change of the O helix from open to closed is triggered. The O helix is marked with red. (c) Crystal structure of hPol β after protein mutation. The mutation site W325C near the O helix is marked with a circle. (d) Protein purity analysis by SDS-PAGE. Stocking Buffer: 50mM Tris-HCl (pH = 8.0), 300 mM NaCl, 1 mM DTT, 1 mM EDTA, 50 % (v/v) glycerol. Concentration of protein: 0.6mg/ml. Purify: over 95 %. (e) The molecular weight of hPol *β* characterized by LC-MS was 39485.00, consistent with the target protein. (f) DNA polymerase activity test.

For DNA polymerase activity test (Figure S5f), we designed an experiment that a short primer and a long primer can be annealed and the DNA polymerase can bind and elongate the short primer. Taq DNA polymerase was used as positive control, but it just partially finished the elongation. Analyzed by native PAGE, there were two bands in PC lane: One is the same with NC and the other is like hPol *β*, which completes the elongation. There is only one band in each lane.

A wild-type hPol *β* protein was prepared for enzyme activity comparison. Referring to the method of polymerase steady-state kinetics (Figure S6),[5] the k_{cat} values of WT and W325C with different dNTPs were obtained by using the Michaelis-Menten equation for single nucleotide extension of the primer with one kind of dNTPs (Figure S7). The k_{cat} of the incorporation of dATP，dCTP，dGTP，and dTTP by hPol *β* WT and W325C was showed in Table S1. From the table, we can see that the k_{cat} of polymerase hPol *β* slightly increased after the mutation of W325 to cysteine, indicating that the mutation does not reduce the enzyme activity and, on the contrary, it will increase the enzyme activity slightly. A DNA template prime as shown below was used in the experiment.

5’ FAM–TCGTGAGCGTCGT–3'

5’ –ACATGAGTCGGT–3'

5’ –ACCGACTCATGTAACGACGCTCACGA–3'

5’ –ACCGACTCATGTCACGACGCTCACGA–3'

5’ –ACCGACTCATGTGACGACGCTCACGA–3'

5’ –ACCGACTCATGTTACGACGCTCACGA–3'

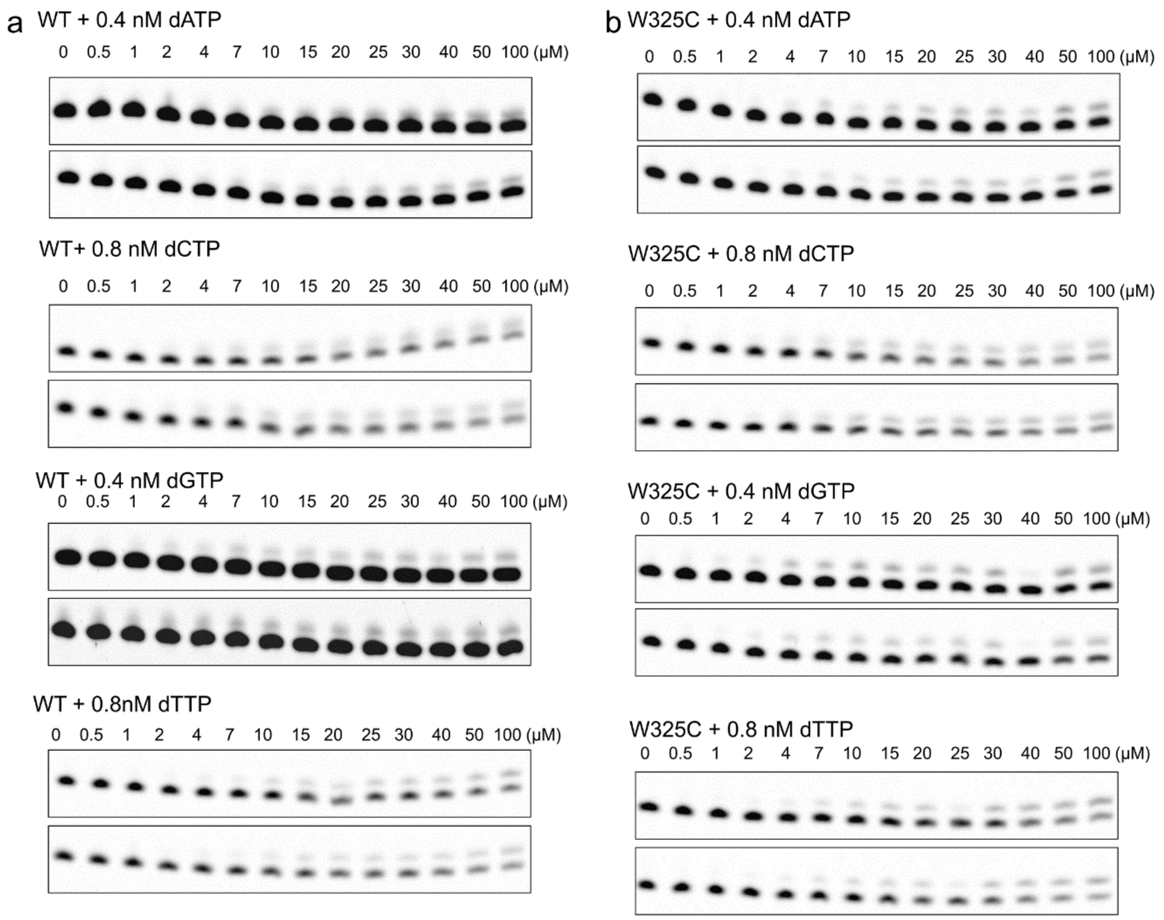

Figure S6. Assay enzyme activity comparison of single nucleotide extension. (a) WT and (B) W325C.

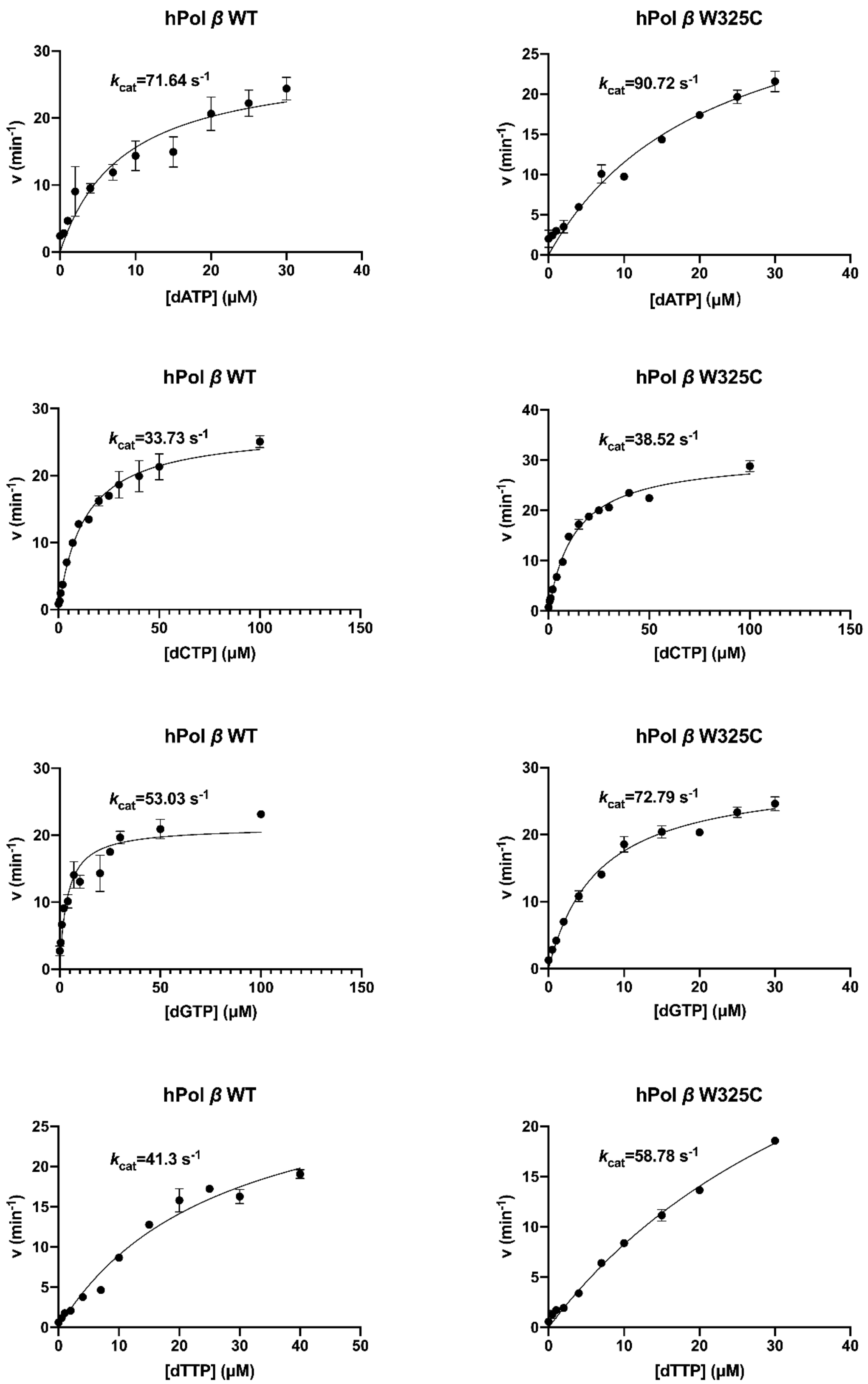

Figure S7. k_{cat} values of WT and W325C with different dNTPs. k_{cat} values for single nucleotide extension were calculated from the Michaelis-Menten equation.

Table S1. k_{cat} **of the incorporation of dATP，dCTP，dGTP，and dTTP by hPol** β **WT and W325C.**

	dATP	dCTP	dGTP	dTTP
WT	71.64 s^{-1}	33.73 s^{-1}	53.03 s^{-1}	41.30 s^{-1}
W325C	90.72 s^{-1}	38.52 s^{-1}	72.79 s^{-1}	58.78 s^{-1}

Section 6. Electrical characterization and control experiments

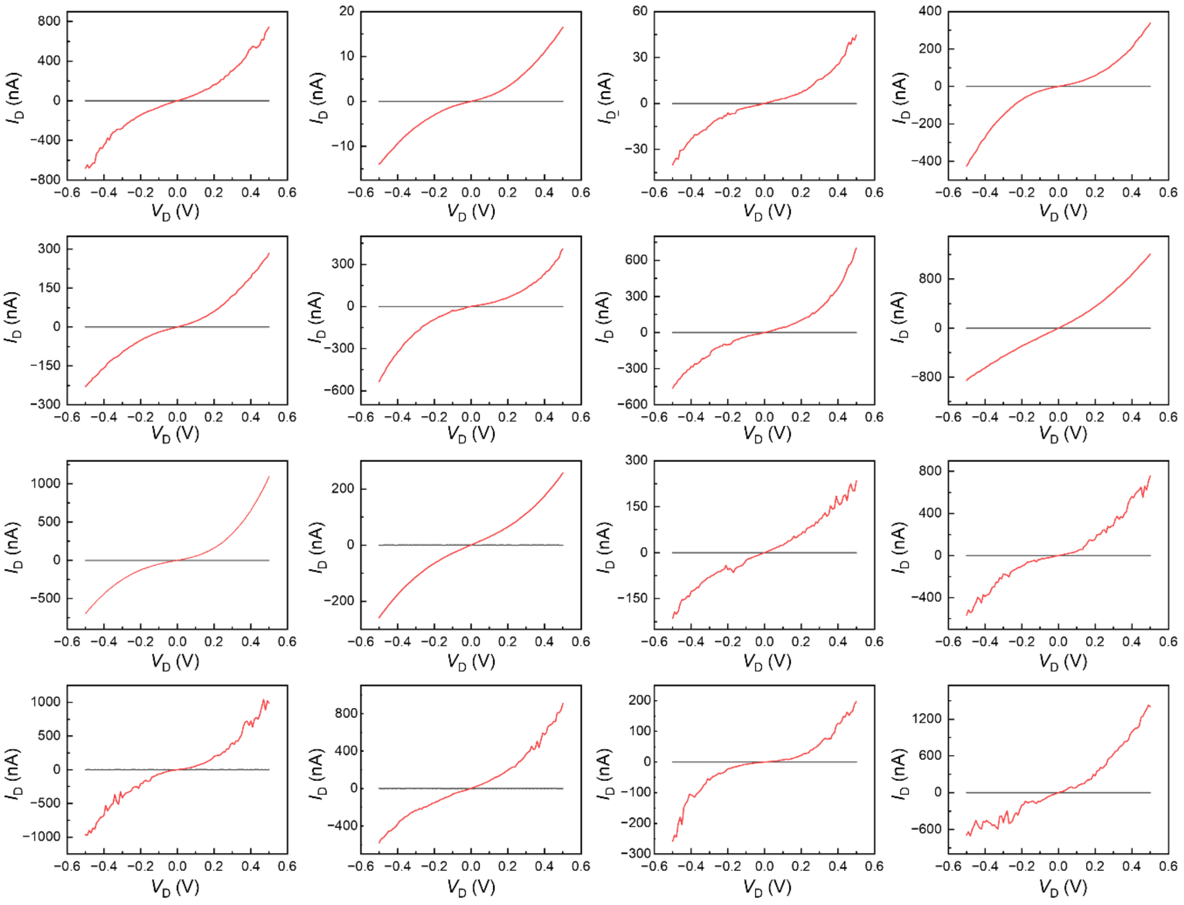

Figure S8. *I*−*V* curves of other 16 GMG single-molecule bridges. Black lines represent open circuits and red lines represent the working GMG single-molecule bridges.

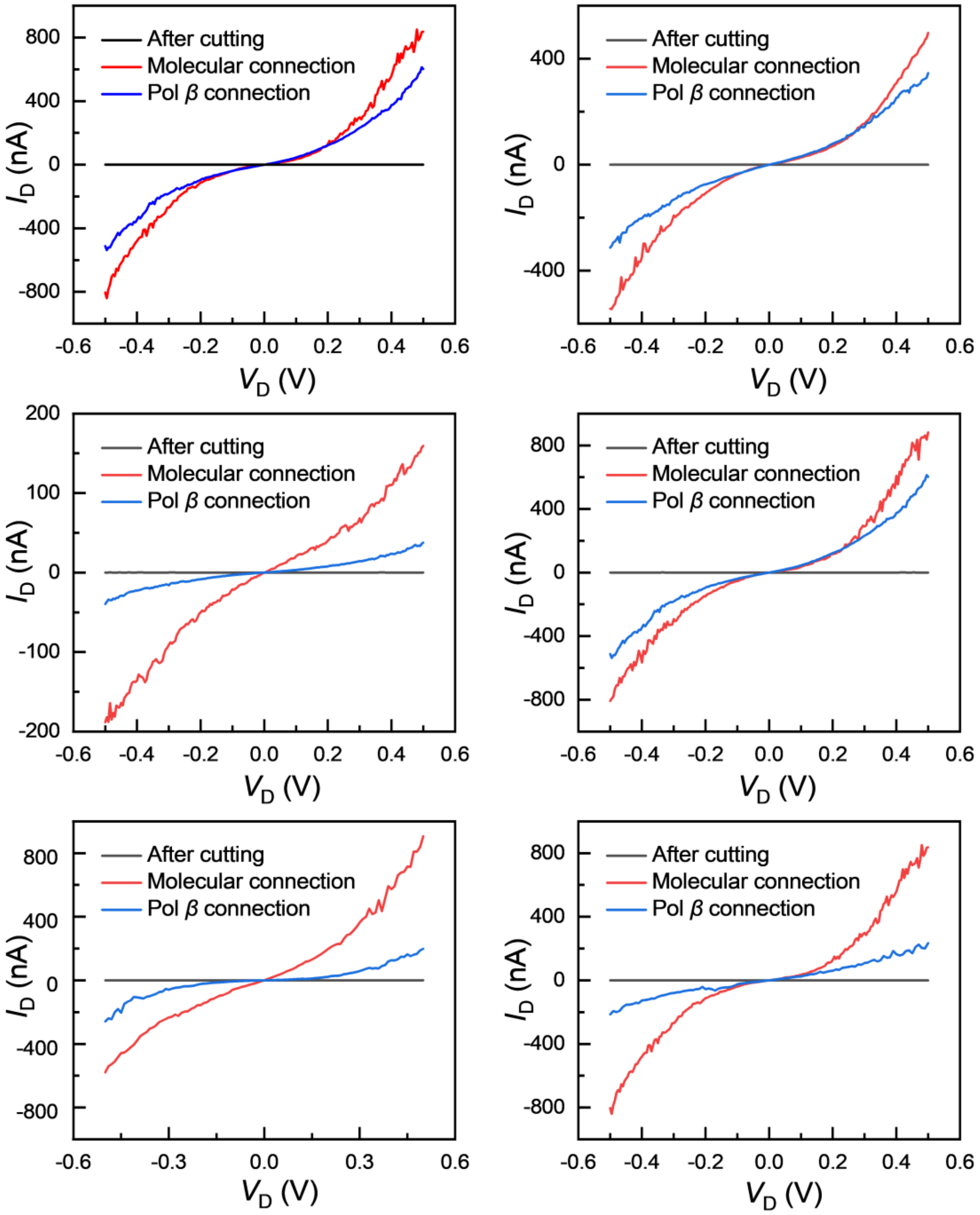

Figure S9. Electrical properties of 6 single-molecule devices. *I–V* curves of the open circuit with graphene point contacts (black), the single-molecular junction (red), and the single-protein junction through covalent connection on the single maleimide bridge (blue).

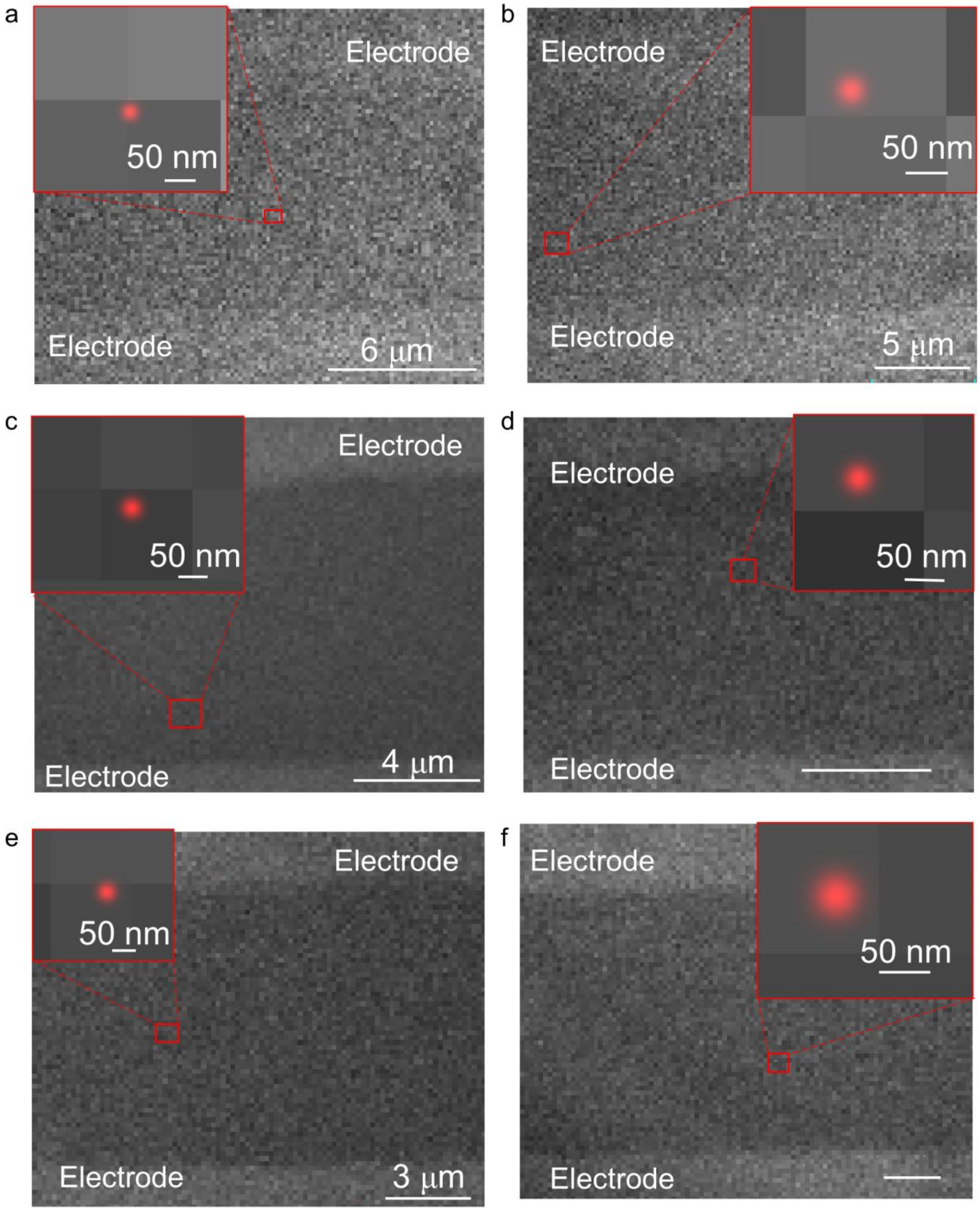

Figure S10. Superhigh-resolution images of 6 different single-protein devices characterized by stochastic optical reconstruction microscopy (STORM). All the measured 6 devices (a−f) showed only single-protein integration on single-maleimide junctions.

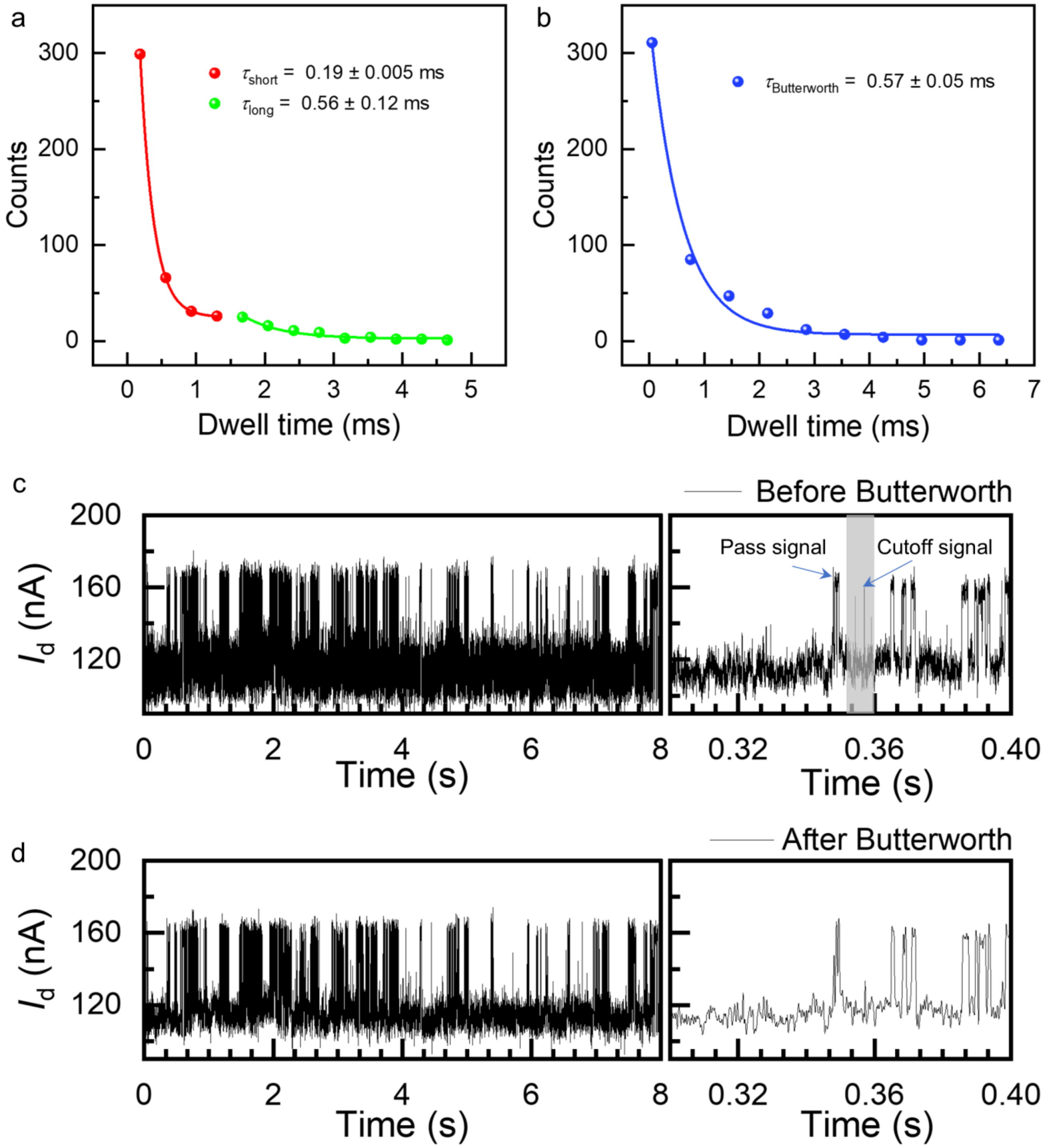

Figure S11. Comparison of dwell time distributions and electrical signals before and after Butterworth filtering of *I*−*t* curves. (a) The dwell time distributions obtained from the raw data. (b) the dwell time distributions after Butterworth filtering. (c) the data before filtering. (d) the data after filtering.

The dwell time distributions, obtained from the raw data, obviously cannot be fitted with a single exponential decay function (Fig. S11A). However, when the dwell time distributions were divided into two categories, they were all fitted well by a single exponential decay function. A short dwell time (τ_{short}) about 0.19 ms and a long dwell time (τ_{long}) about 0.56 ms were obtained. In addition, the dwell time distributions, after Butterworth filtering, were fitted well by a single exponential decay function (Figure S11b). Comparing the data before and after filtering (Figure S11c–d), two electrical signal modes were distinguished. One is the low-pass-filtered binary switch cut-off signal (gray shade) and the other is the low-pass-filtered pass signal corresponding to relatively slow dNTP incorporation into DNA.

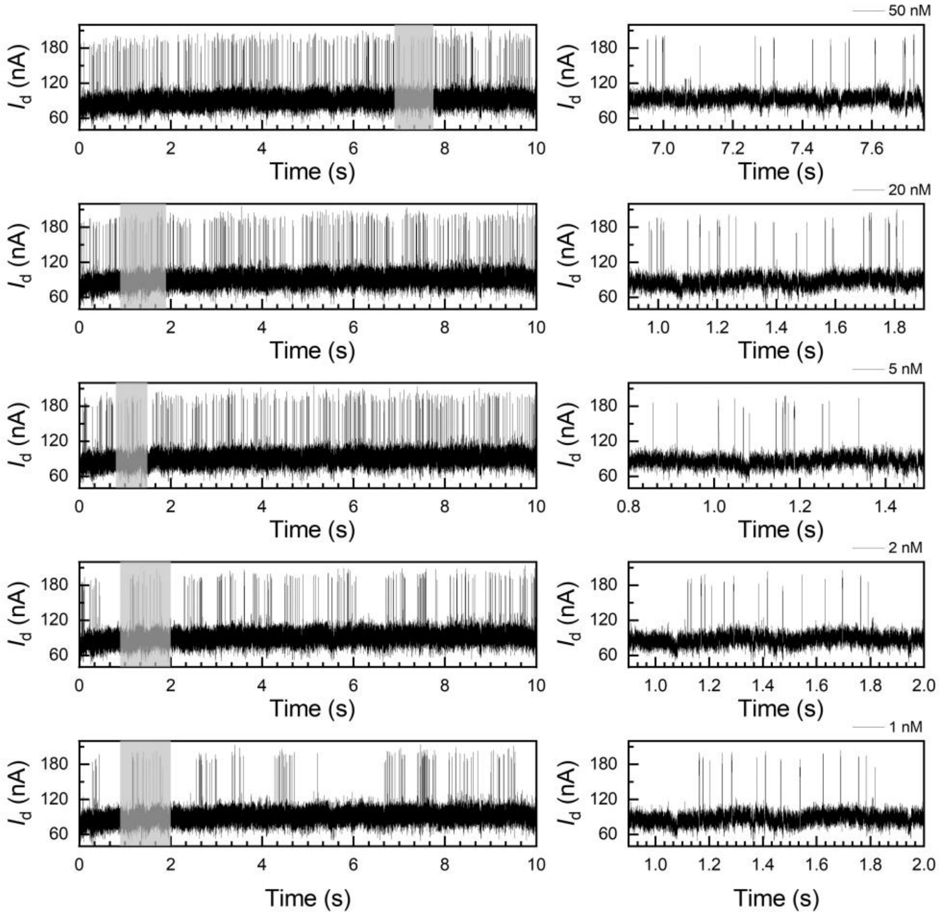

Figure S12. *I–t* curves of the reaction system at low DNA template concentrations (50 nM, 20 nM, 5 nM, 2 nM, and 1 nM).

To demonstrate that DNA polymerase is inserting for each conformational change, we recorded *I–t* curves of the reaction system at low template concentrations (50 nM, 20 nM, 5 nM, 2 nM, and 1 nM) as shown in Figure S12. In this assay of the DNA template prime as shown below, after the addition of dTTP, the following reaction occurs, where 15 T are the newly incorporated dTTP.

3' –ACATTTTGCTGCCGGTCA AAAAA AAAAA AAAAA–5'

5' –TGTAAAACGACGGCCAGT–3' + dTTP ⟶

3' –ACATTTTGCTGCCGGTCA AAAAA AAAAA AAAAA–5'

5' –TGTAAAACGACGGCCAGT TTTTT TTTTTT TTTTTT–3' + PP_i

Interestingly, with the concentration decrease, the electrical signals scattered or clustered signals appeared at low concentrations. Moreover, when the template concentration is below 2 nM, the signal variation number of every clustered signal is consistent with the number of newly incorporated bases, demonstrating that DNA polymerase is inserting for each current signal variation.

Moreover, considering the average number of bases incorporated by the DNA polymerase during a single DNA binding event, which is about 20 for DNA Polymerase *β*,[6] the DNA template prime we selected is only 15 in length.

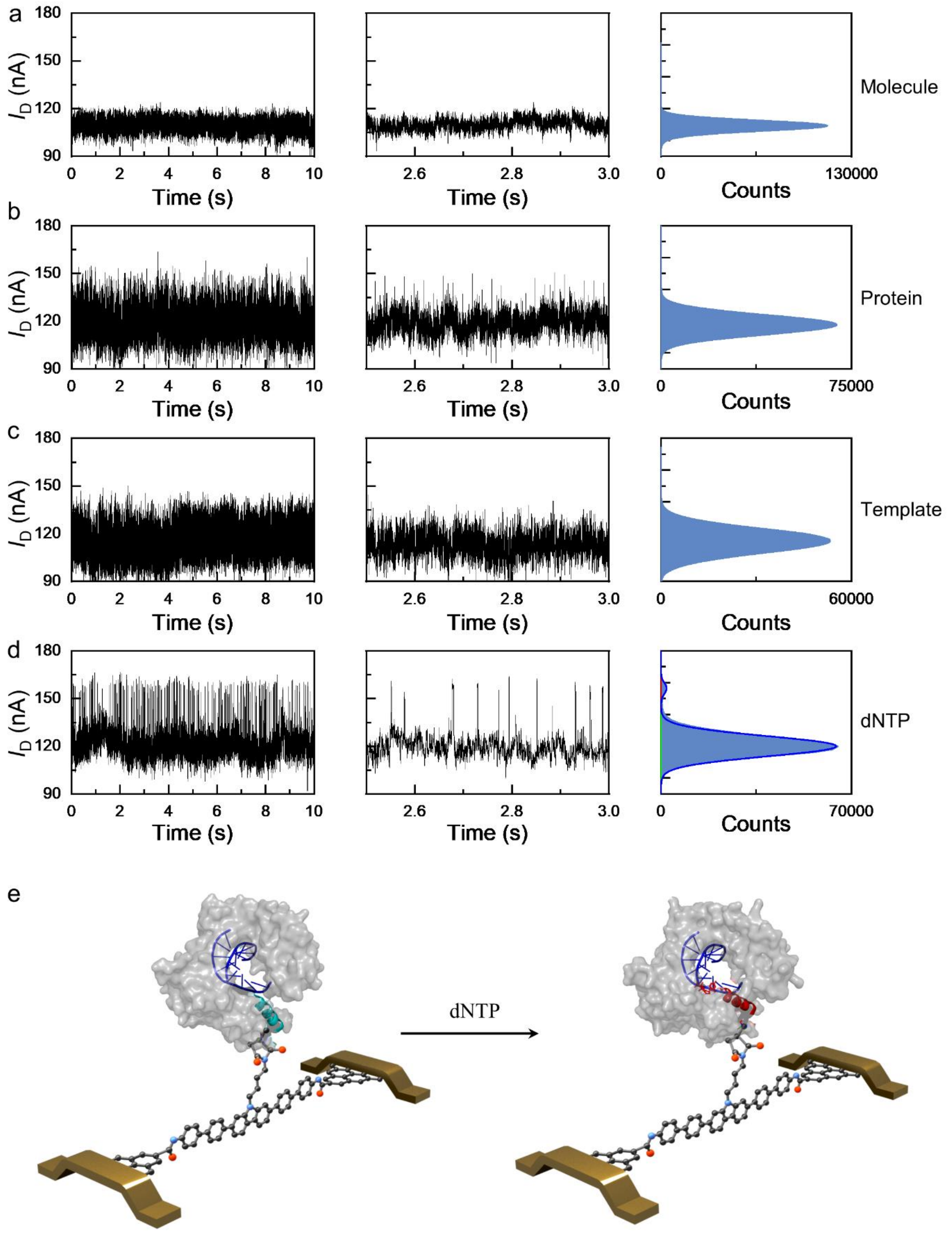

Figure S13. Electrical characterization and signal attribution of single-molecule DNA synthesis by catalyzed hPol *β*. Current signal variations with a bias voltage of 200 mV between source and drain electrodes during the successive addition of the reaction substrates at 37 °C. The middle panels show the 0.5 s magnified view of each current curve and the right panels are the corresponding current histograms of each current curve. (a) Molecule; (b) Protein; (c) DNA template. The conductance stays in the open state. (d) dNTP solution. A new conductance state with the current level at ~150 nA appeared and the two conducting states occurred alternately. (e) Schematic diagram of the conformational change of the O helix from open (blue) to closed (red) for each catalytic cycle. Attributions of the two conductance states in the current signal from low to high are open and closed states of hPol *β*.

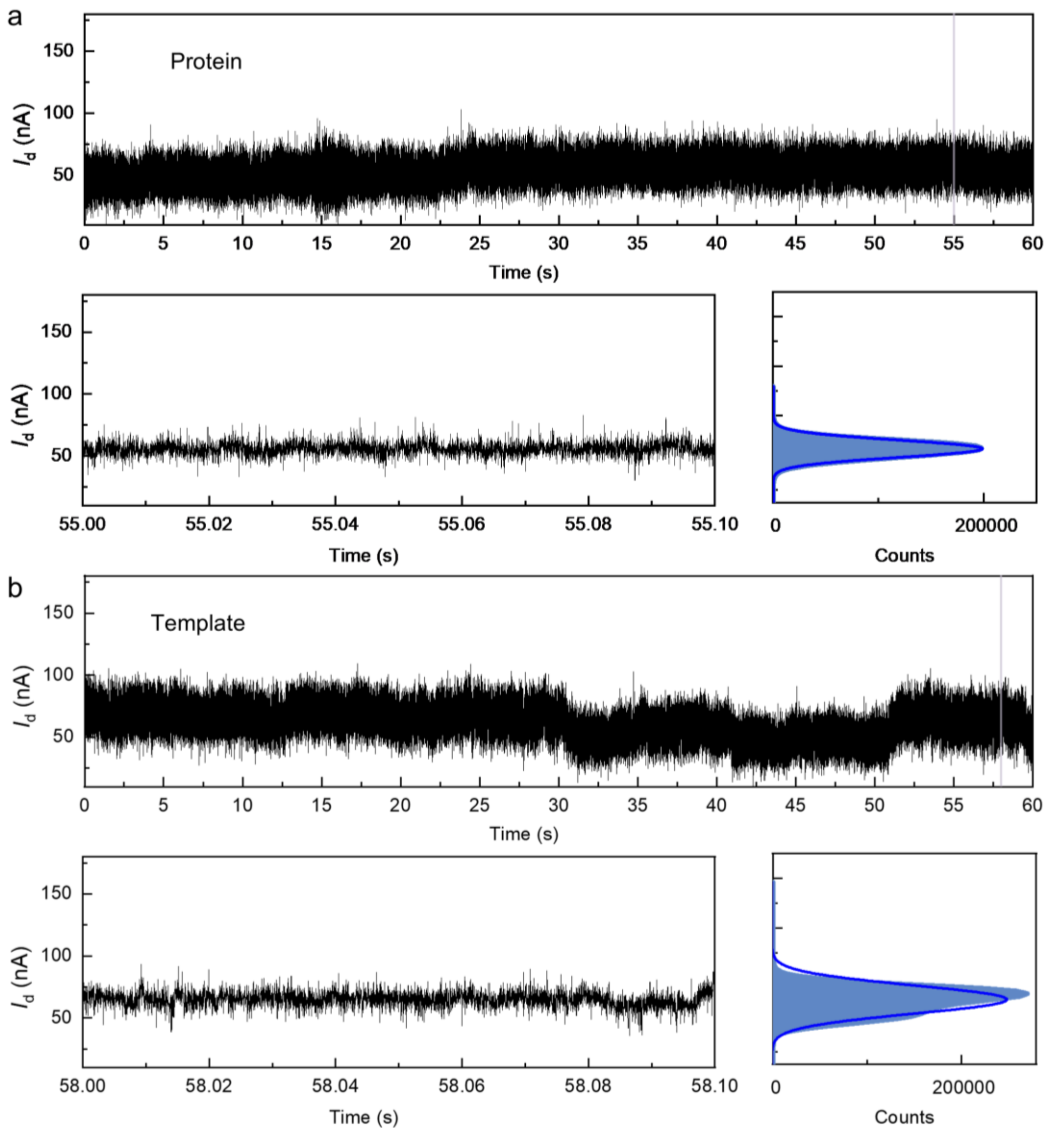

Figure S14. Continuous control measurements at 37 °C for 60 s without the introduction of DNA (a) and without the introduction dNTPs (b). *I–t* curves at 200 mV, magnification diagram for 1 s, and corresponding statistical histogram. No obvious fluctuations in the *I–t* curves and the single peak in the Gaussian fitting indicate the reliability of our devices.

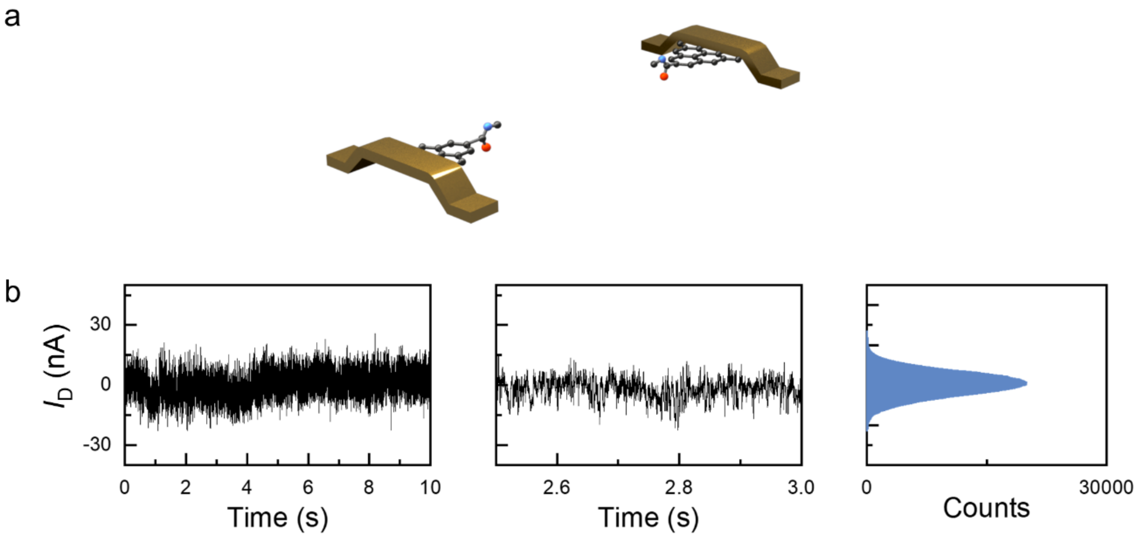

Figure S15. Characterization of an open circuit device at 37 ºC. (a) Schematic diagram of an open circuit device. (b) *I*–*t* curves at 200 mV and corresponding statistical histogram of the electrical monitoring platform before connection of the molecular bridge (open circuit). No obvious fluctuations in the *I*–*t* curves and the single peak in the Gaussian fitting indicate stable electrodes.

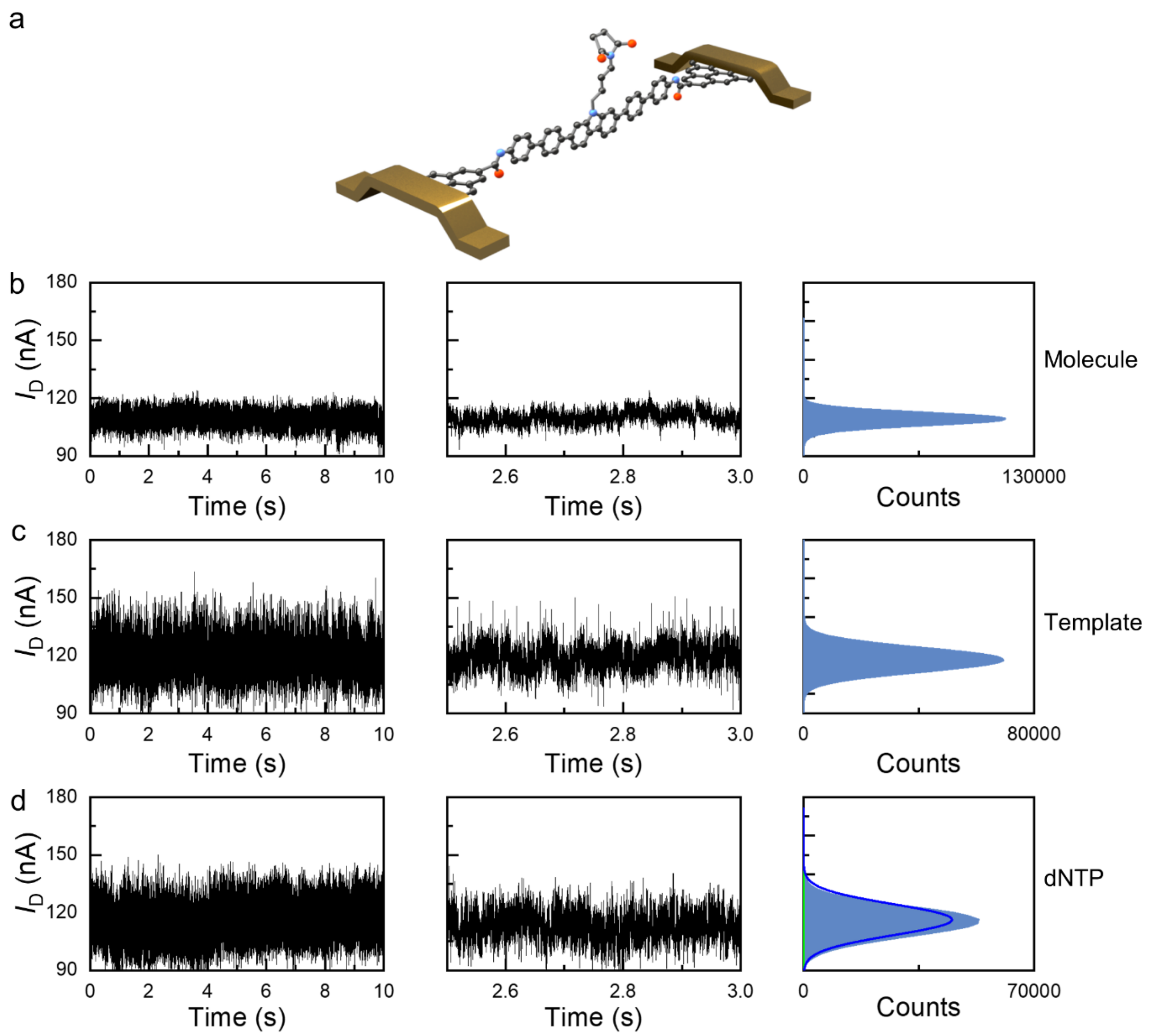

Figure S16. Characterization of a single-molecule device formed from the molecular bridge. (a) Schematic diagram of the single-molecule device. Current signal variations with a bias voltage of 200 mV between source and drain electrodes during the successive addition of the reaction substrates at 37 °C. The middle panels show the 0.5 s magnified view of each current curve and the right panels are the corresponding current histograms of each current curve. (b) *I*–*t* curves in buffer solution (Molecule). (c) *I*–*t* curves after the addition of DNA template. (d) *I*–*t* curves after further introduction of dNTP. No obvious fluctuations in the *I*–*t* curves and the single peak in the Gaussian fitting were found. The change of the molecular configuration did not result in the obvious changes of the conductance state.

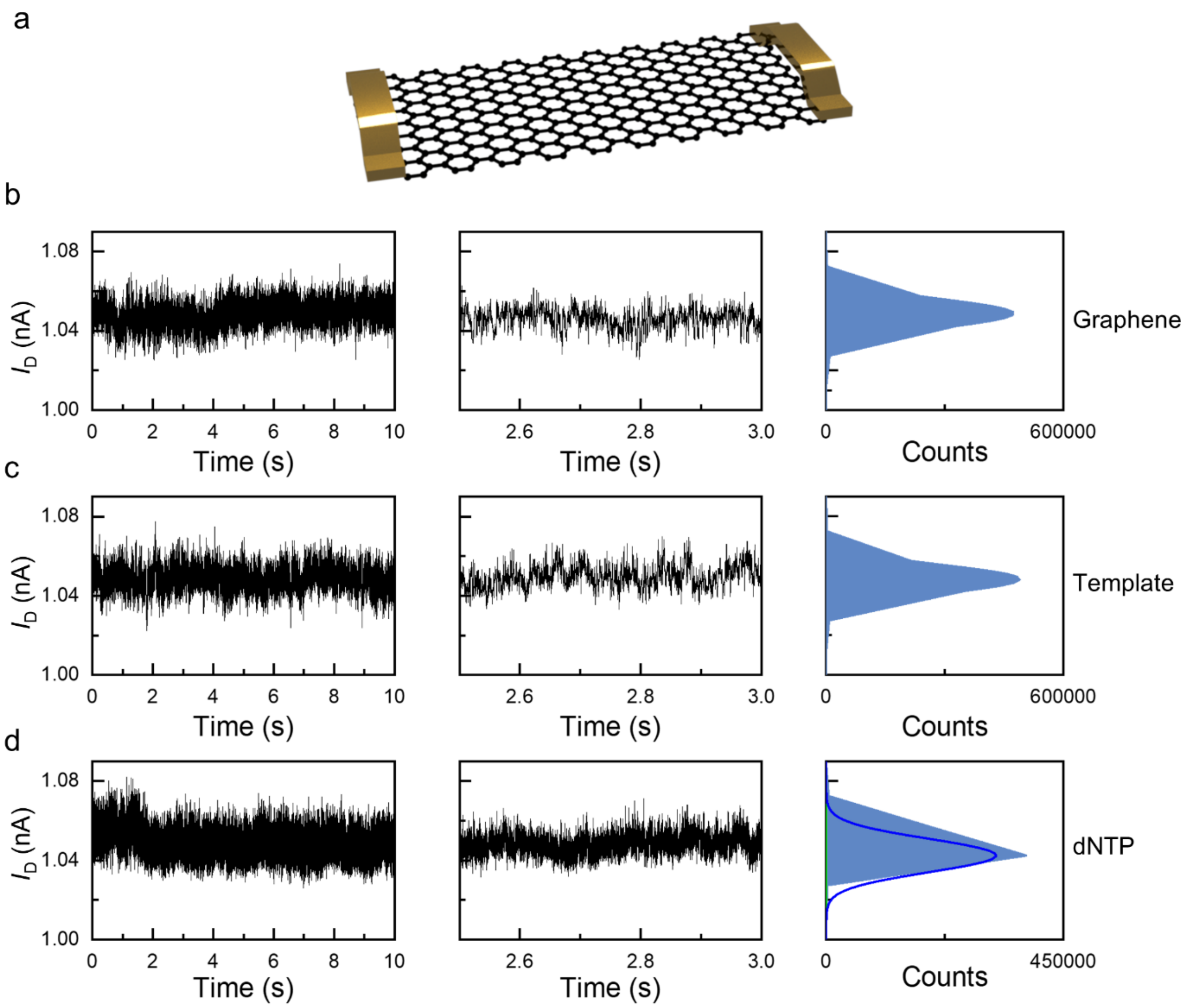

Figure S17. Characterization of a graphene nanoribbon device. (a) Schematic diagram of the graphene nanoribbon device. Current signal variations with a bias voltage of 200 mV between source and drain electrodes during the successive addition of the reaction substrates at 37 °C. The middle panels show the 0.5 s magnified view of each current curve and the right panels are the corresponding current histograms of each current curve. (b) *I*–*t* curves in buffer solution. (c) *I*–*t* curves after the addition of DNA template. (d) *I*–*t* curves after further introduction of dNTP. We found no significant changes in the current levels with different reaction substrates.

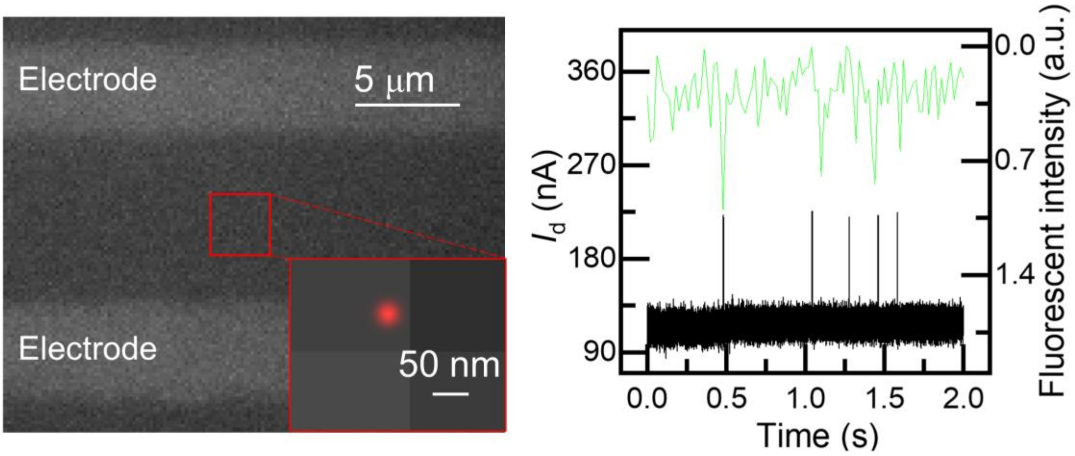

Figure S18. Stochastic optical reconstruction microscopy (STORM) characterization. Simultaneously recorded fluorescent (green) and electrical (black) signals under addition of DNA template and cy3-labeled dNTP (γ-[(6-Aminohexyl)-imido]-dATP-Cy3) match each other very well. arb. units: arbitrary units. Bias voltage 200 mV, temperature 37 °C.

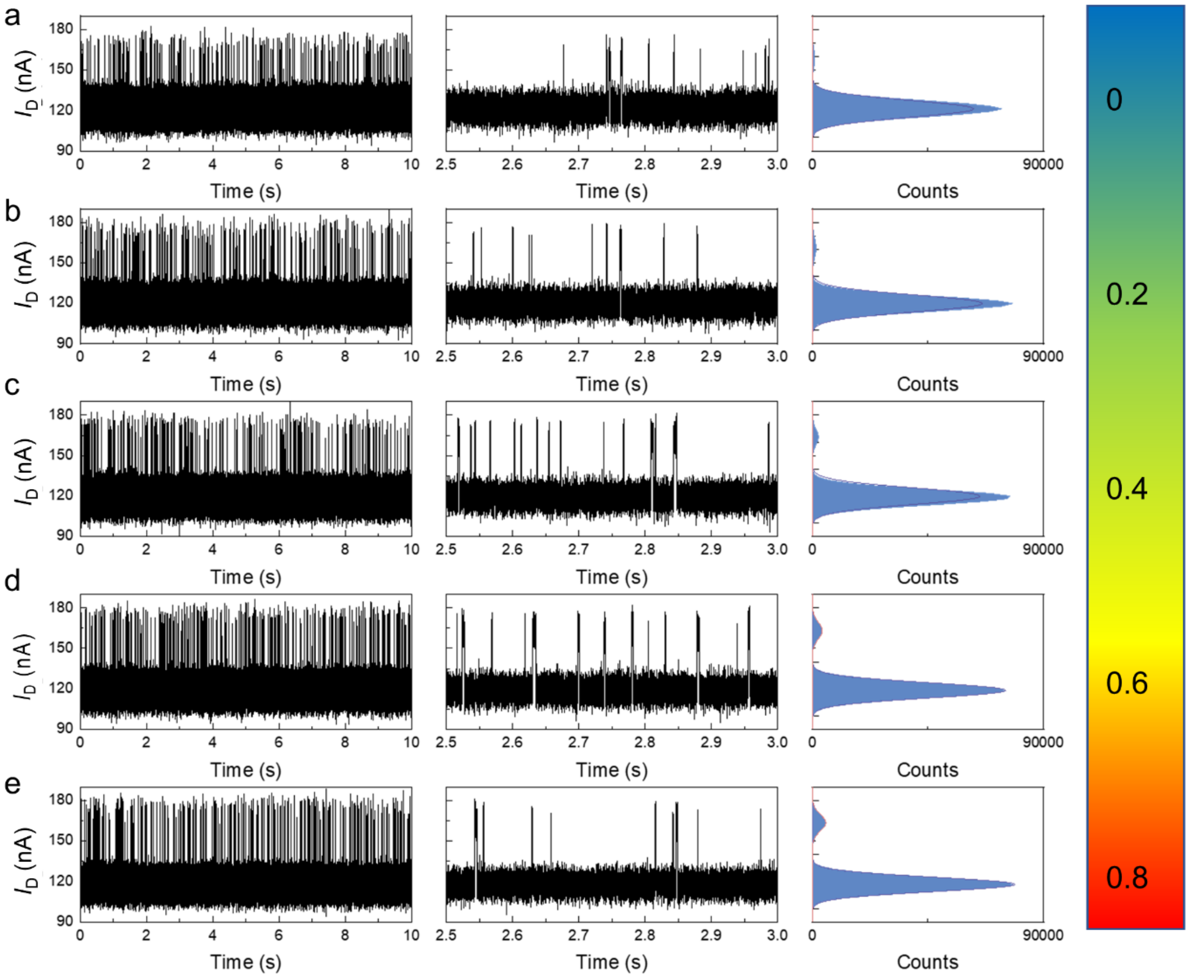

Figure S19. Isotope effect measurements at 37 °C and 200 mV. Concentration-dependent measurements at various D_2O contents: *I*–*t* curves (10 s) in five mole fractions of D_2O (a) 0, (b) 0.2, (c) 0.4, (d) 0.6, and (e) 0.8 at 200 mV. The middle panels show the 0.5 s magnified view of each current curve and the right panels are the corresponding current histograms of each current curve. The *I*–*t* curves show that as the fractions of D_2O increased, the dwell times of high conductivity state gradually increased.

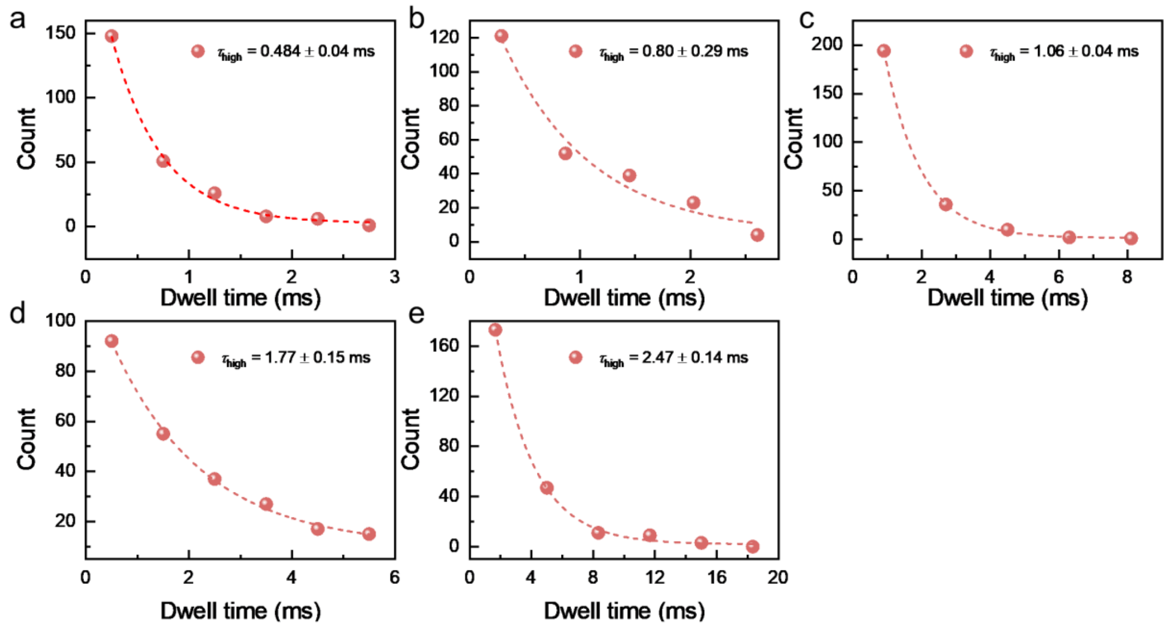

Figure S20. Statistical analysis of the dwell times belonging to the high state (red) in five mole fractions of D_2O (a) 0, (b) 0.2, (c) 0.4, (d) 0.6, and (e) 0.8 at 200 mV.

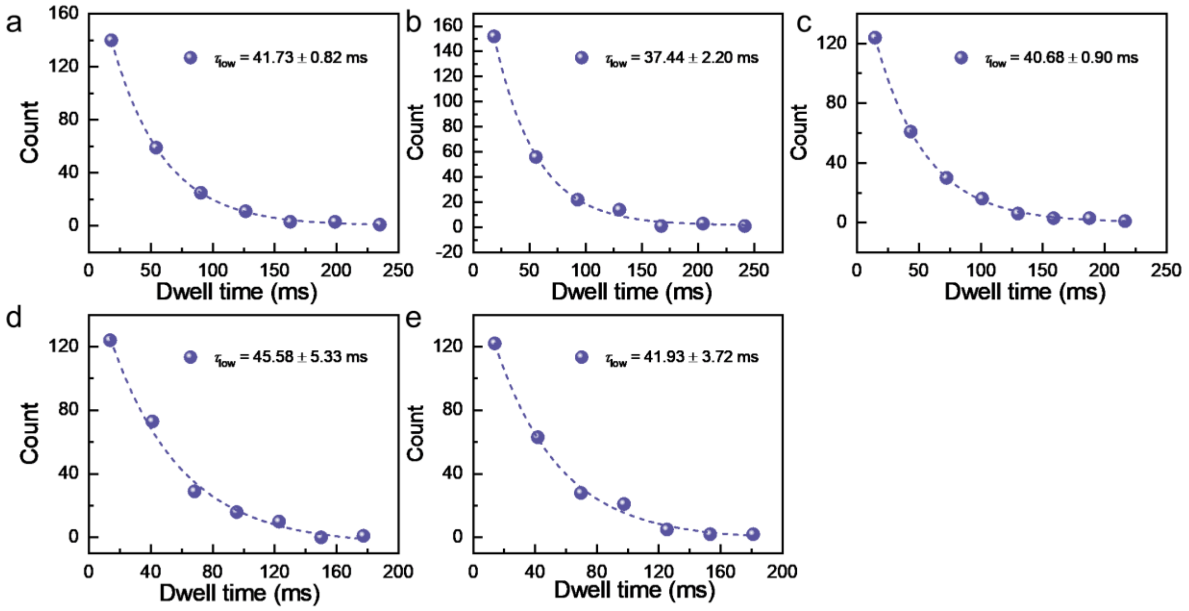

Figure S 21. Statistical analysis of the dwell times belonging to the low state (blue) in five mole fractions of D_2O (a) 0, (b) 0.2, (c) 0.4, (d) 0.6, and (e) 0.8 at 200 mV.

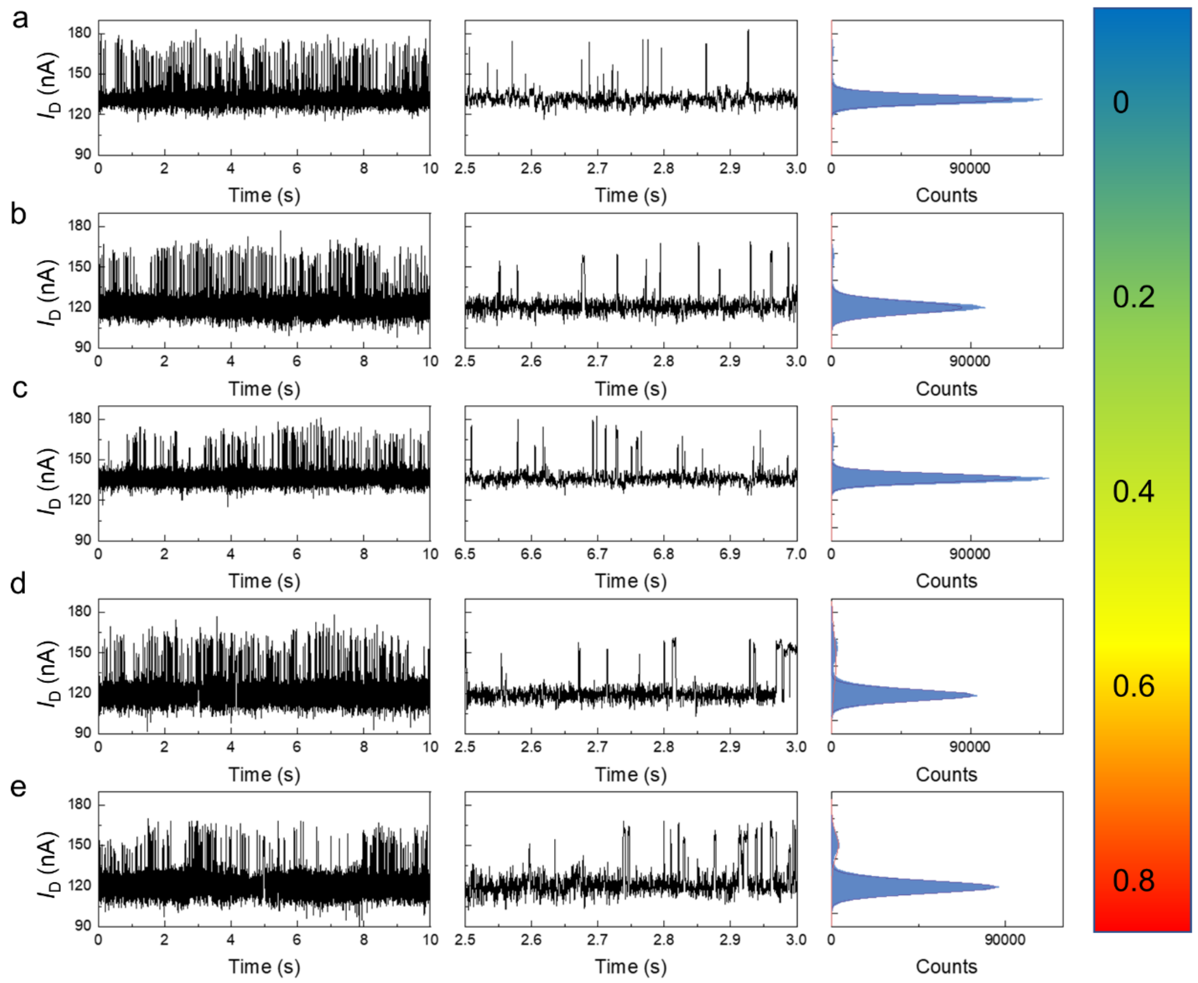

Figure S22. Other isotope effect measurements at 37 °C and 200 mV. Measurements conducted using another single-molecule protein catalyst device with similar results to the previous data in five mole fractions of D_2O: (a) 0, (b) 0.2, (c) 0.4, (d) 0.6, and (e) 0.8.

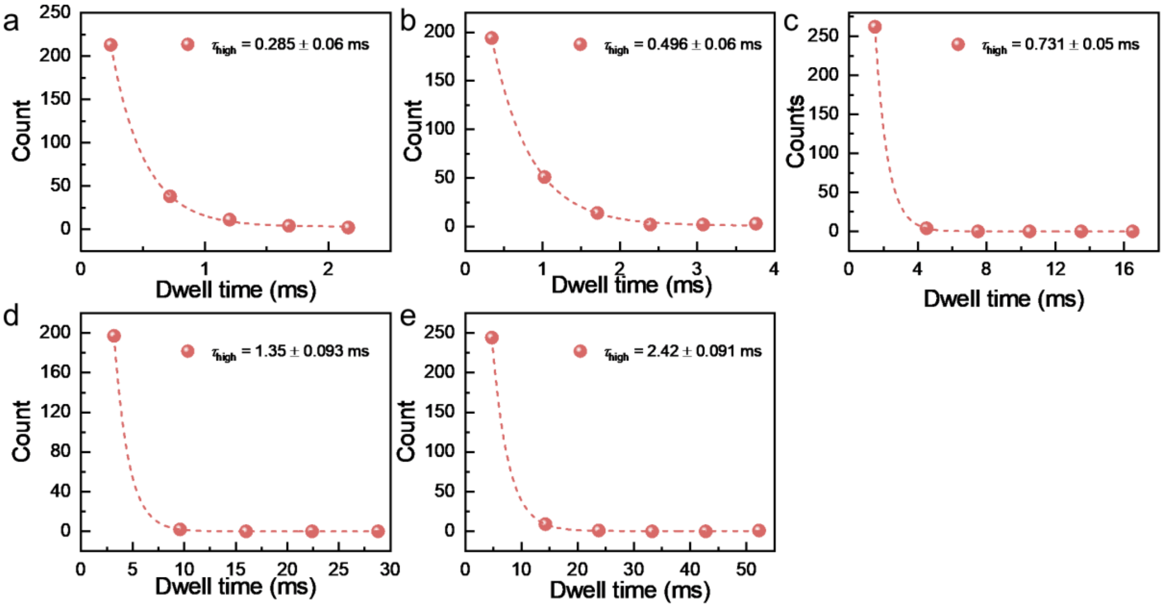

Figure S23. Statistical analysis of the dwell times of the other isotope effect measurements belonging to the high state (red) in five mole fractions of D_2O (a) 0, (b) 0.2, (c) 0.4, (d) 0.6, and (e) 0.8 at 200 mV.

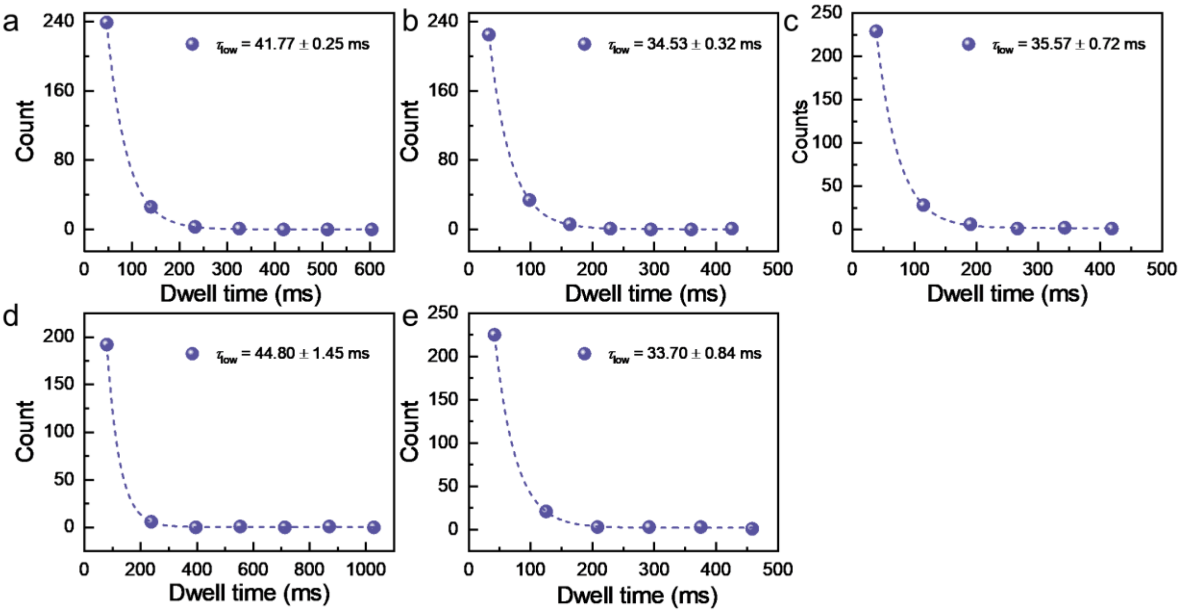

Figure S24. Statistical analysis of the dwell times of the other isotope effect measurements belonging to the low state (blue) in five mole fractions of D_2O (a) 0, (b) 0.2, (c) 0.4, (d) 0.6, and (e) 0.8 at 200 mV.

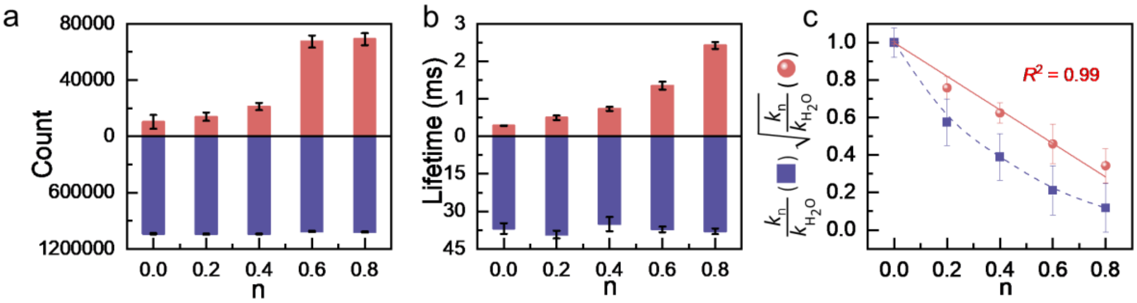

Figure S25. Summary of isotope effect measurements. (a) Occurrence proportion distributions of the open state (top) and the close state (below). (b) The lifetime of the open complex (top) and the close complex (below). (c) Two protons are transferred during nucleotidyl transfer. k_n is the observed rate constant for nucleotide incorporation at a particular mole fraction of D_2O.

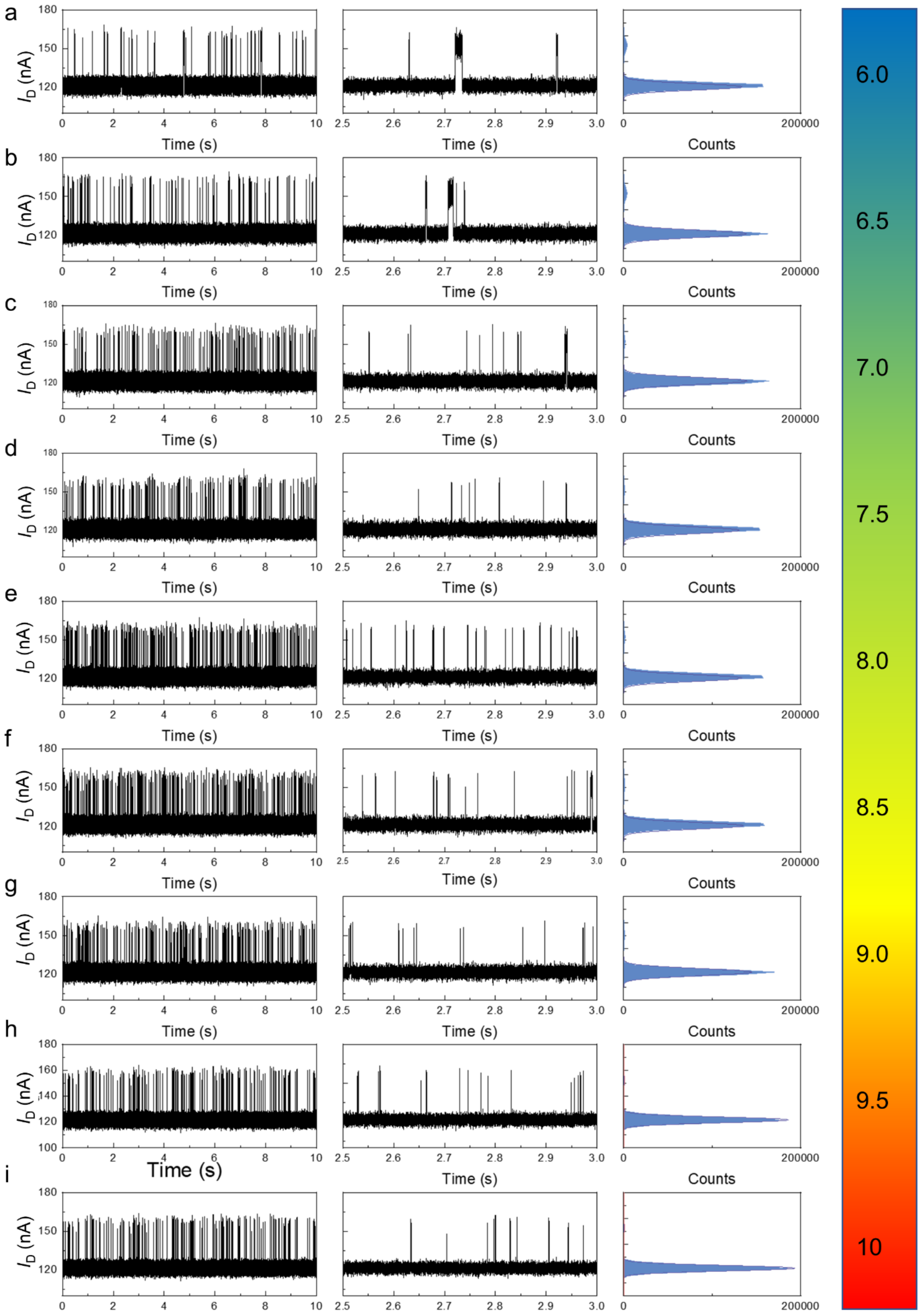

Figure S26. pH dependence for nucleotidyl-transfer reaction at 37 °C and 200 mV: *I*–*t* curves (10 s) in nine pHs between 6.0 and 10.0 in Mg^{2+}: (a) 6.0, (b) 6.5, (c) 7.0, (d) 7.5, (e) 8.0, (f) 8.5, (g) 9.0, (h) 9.5, and (i) 10.0. The middle panels show the 0.5 s magnified view of each current curve and the right panels are the corresponding current histograms of each current curve.

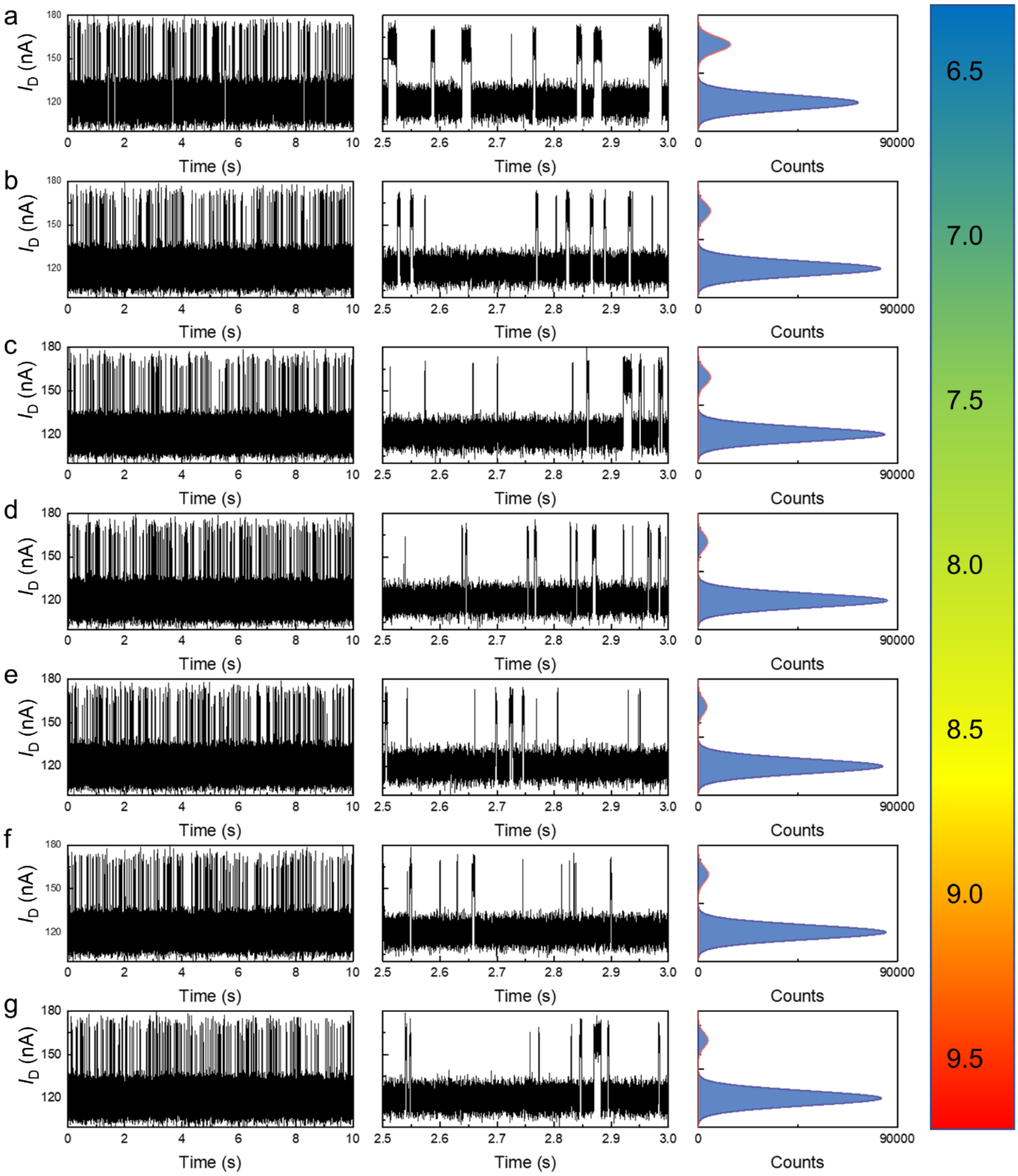

Figure S27. pH dependence for nucleotidyl-transfer reaction at 37 °C and 200 mV: *I*–*t* curves (10 s) in seven pHs between 6.5 and 9.5 in Mn^{2+}: (a) 6.5, (b) 7.0, (c) 7.5, (d) 8.0, (e) 8.5, (f) 9.0, and (g) 9.5. The middle panels show the 0.5 s magnified view of each current curve and the right panels are the corresponding current histograms of each current curve.

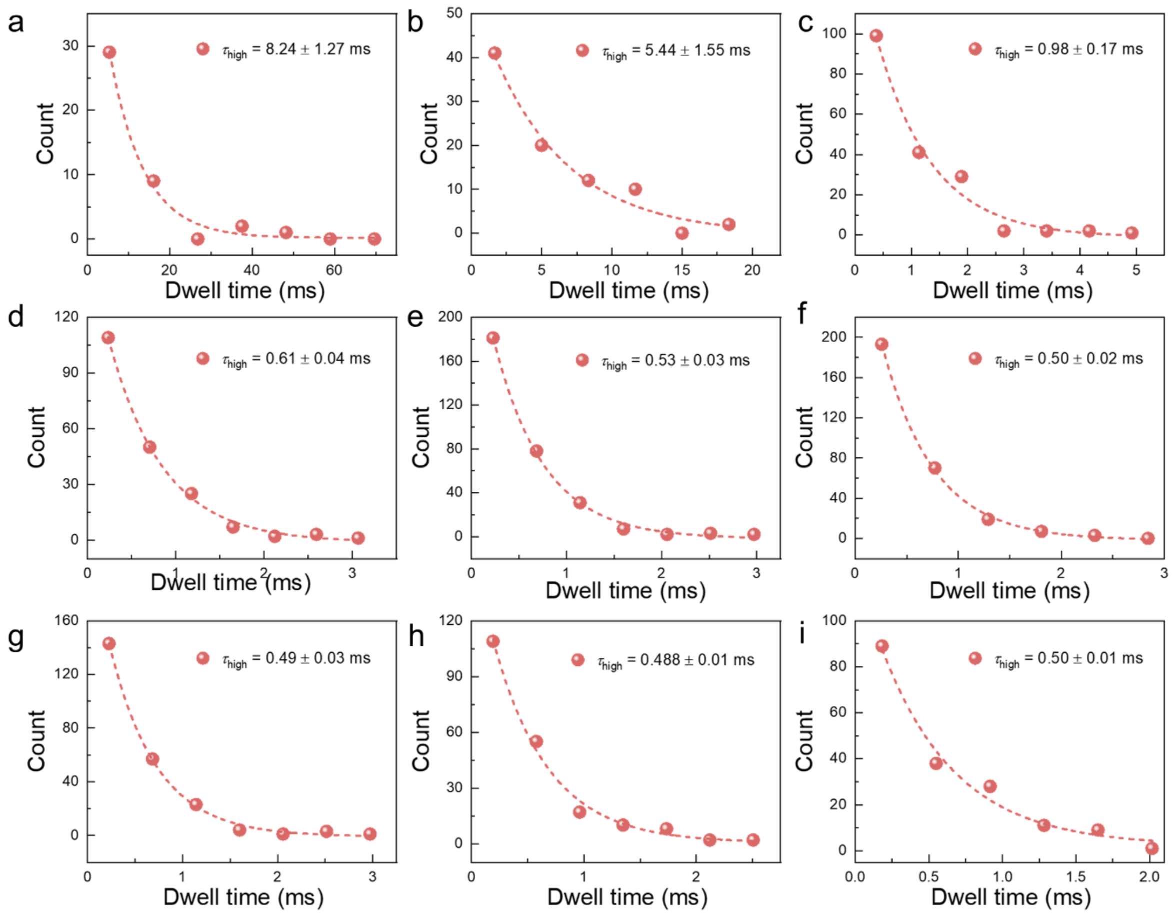

Figure S28. Statistical analysis of the dwell times belonging to the high state (red) in nine pHs between 6.0 and 10.0 in Mg^{2+}: (a) 6.0, (b) 6.5, (c) 7.0, (d) 7.5, (e) 8.0, (f) 8.5, (g) 9.0, (h) 9.5, and (i) 10.0.

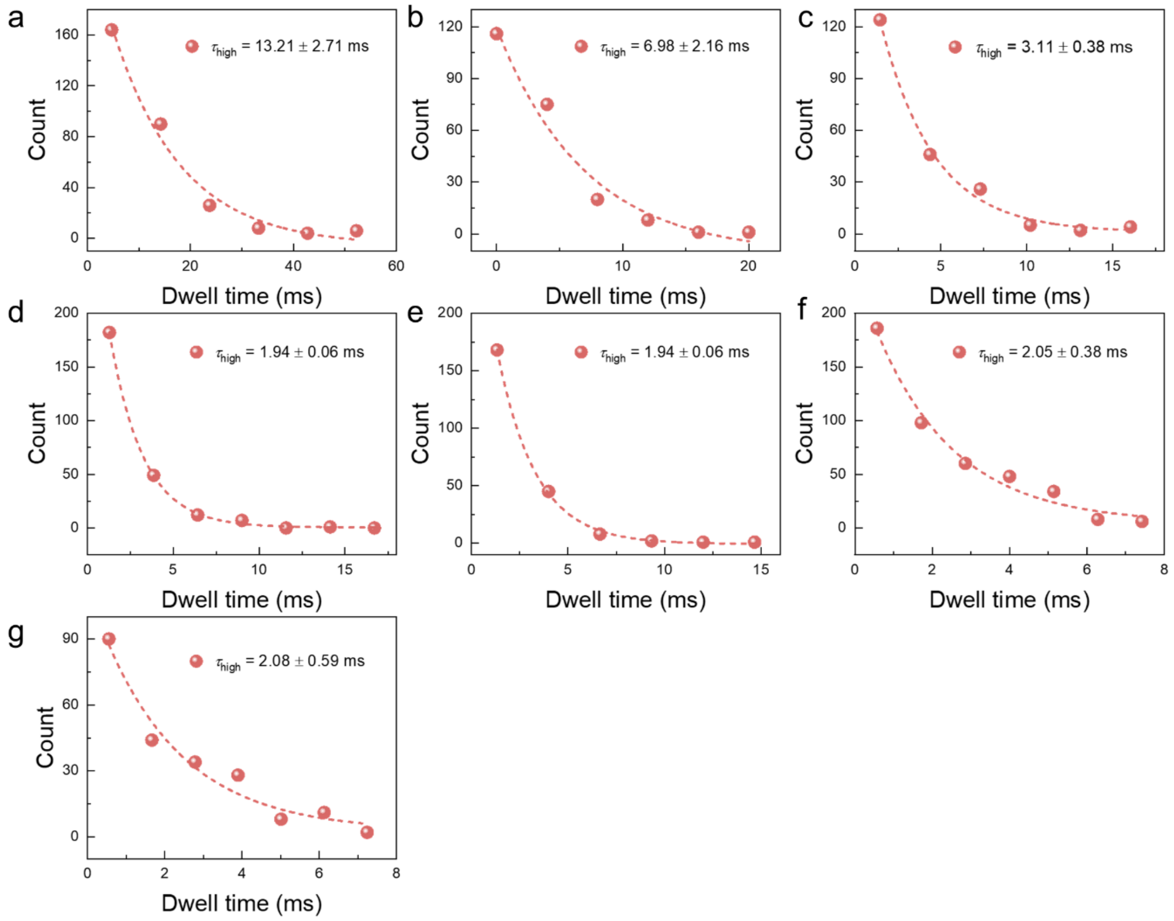

Figure S29. Statistical analysis of the dwell times belonging to the high state (red) in seven pHs between 6.5 and 9.5 in Mn^{2+}: (a) 6.5, (b) 7.0, (c) 7.5, (d) 8.0, (e) 8.5, (f) 9.0 and (g) 9.5.

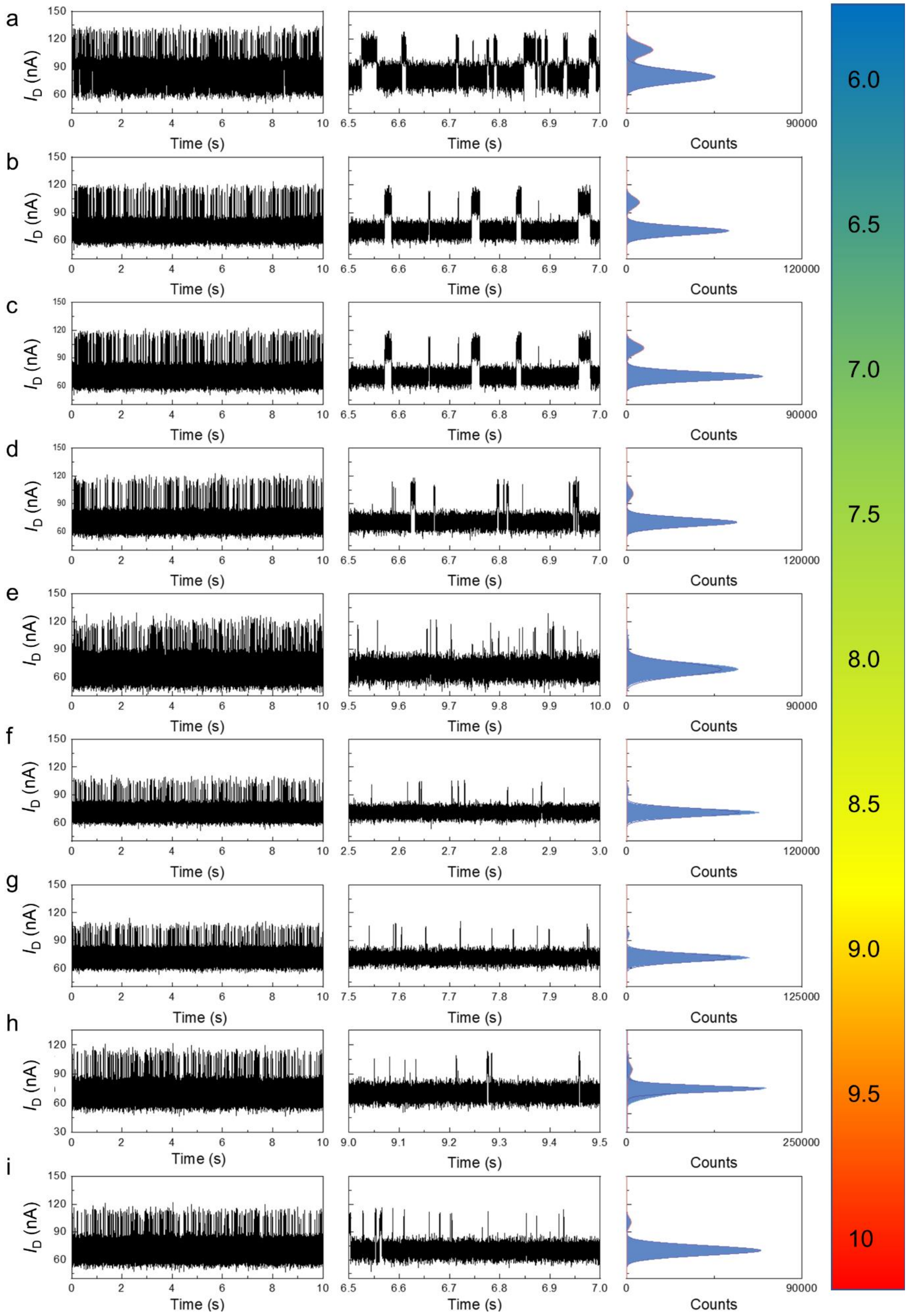

Figure S30. Other pH dependence for nucleotidyl-transfer reaction at 37 °C and 200 mV in Mg^{2+}. Measurements conducted using another single-molecule protein catalyst device shows similar results with the previous data: (a) 6.0, (b) 6.5, (c) 7.0, (d) 7.5, (e) 8.0, (f) 8.5, (g) 9.0, (h) 9.5, and (i) 10.0.

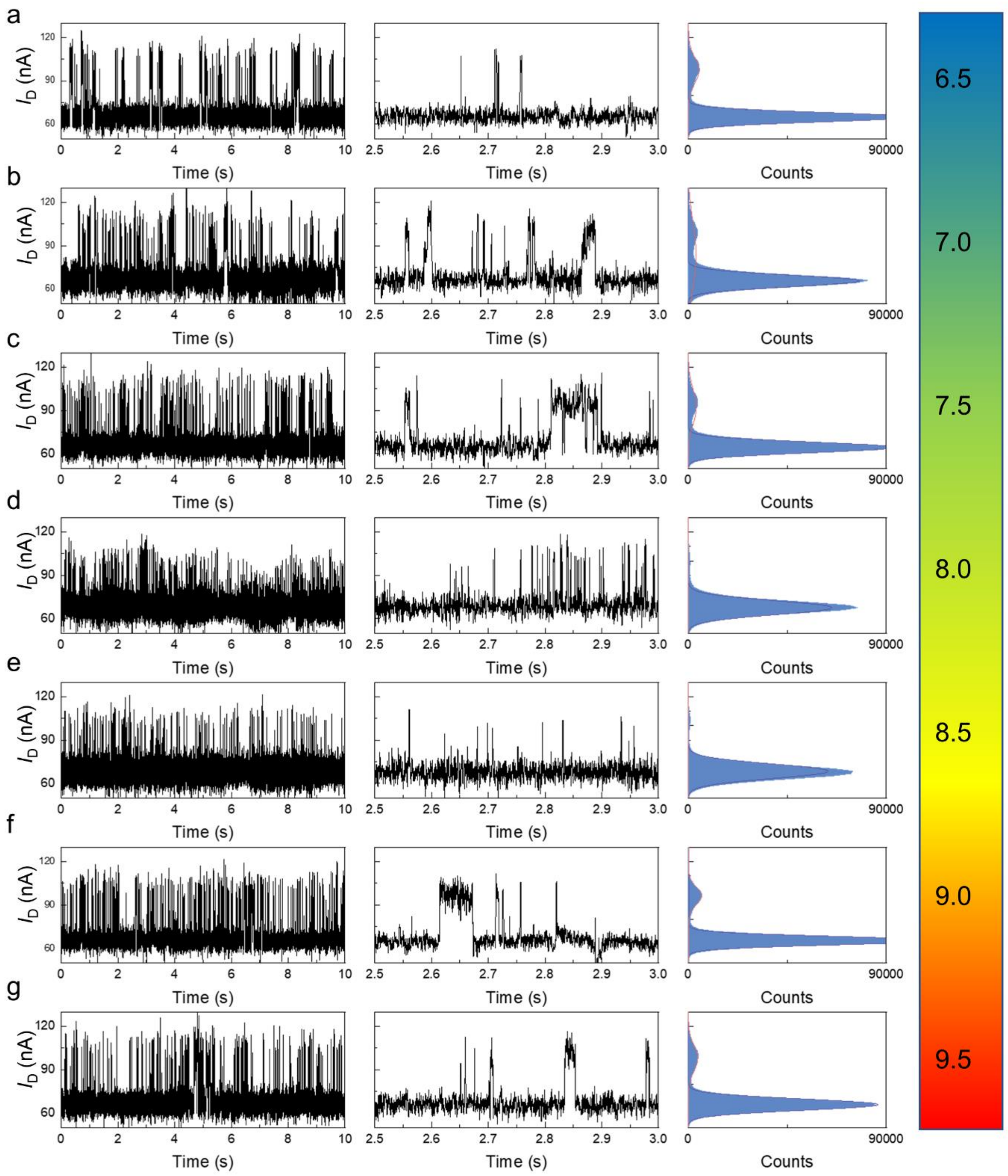

Figure S31. Other pH dependence for nucleotidyl-transfer reaction at 37 °C and 200 mV in Mn^{2+}. Measurements conducted using another single-molecule protein catalyst device shows similar results with the previous data: (a) 6.5, (b) 7.0, (c) 7.5, (d) 8.0, (e) 8.5, (f) 9.0, and (g) 9.5.

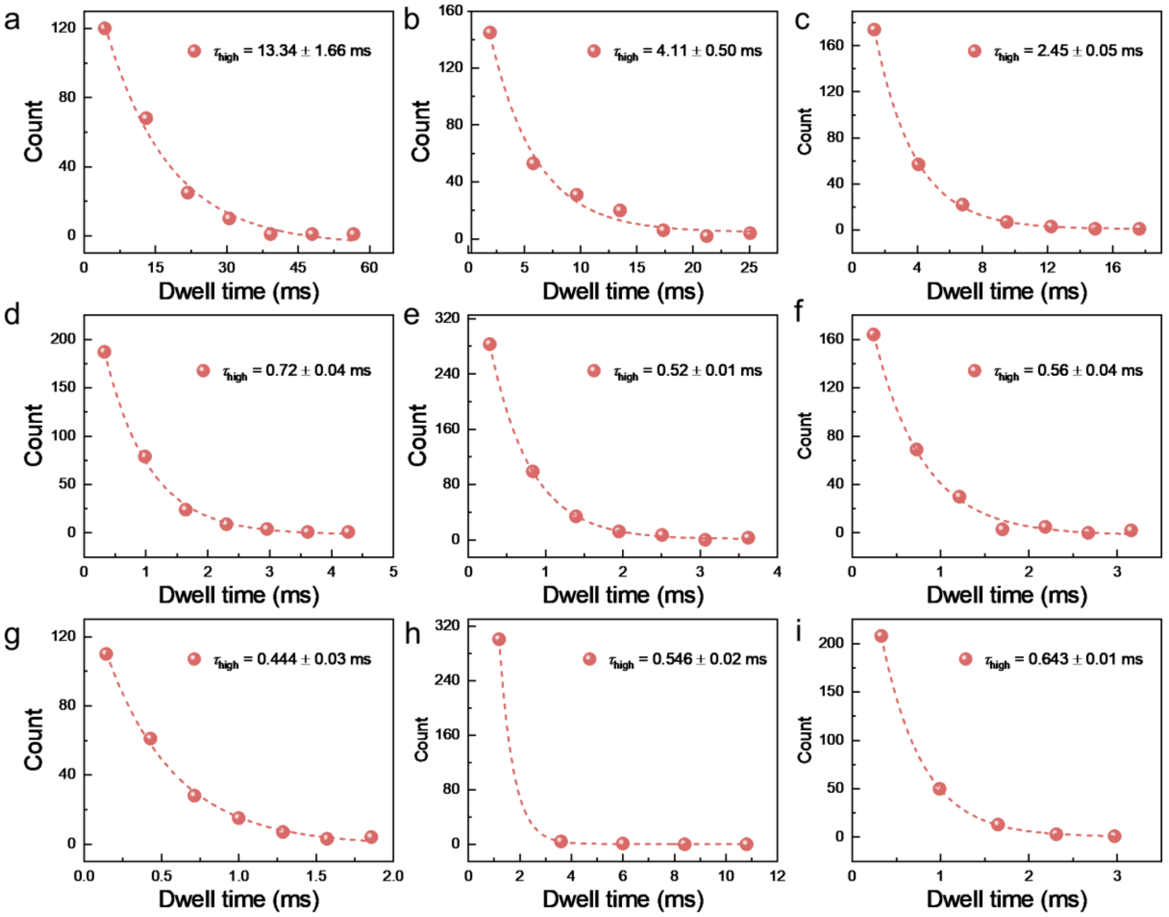

Figure S32. Statistical analysis of the dwell times of the other pH dependence experiment belonging to the high state (red) in nine pHs between 6.0 and 10.0 in Mg^{2+}: (a) 6.0, (b) 6.5, (c) 7.0, (d) 7.5, (e) 8.0, (f) 8.5, (g) 9.0, (h) 9.5, and (i) 10.0.

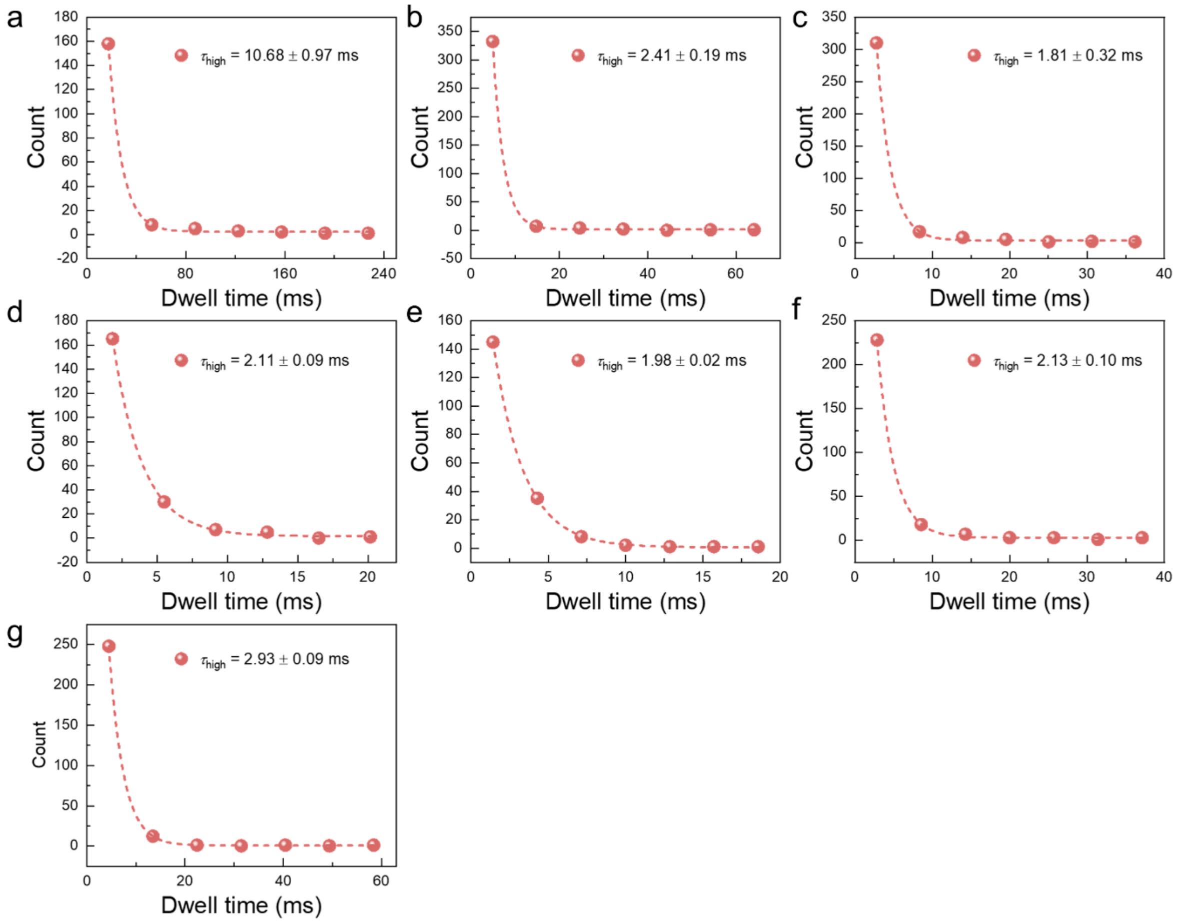

Figure S33. Statistical analysis of the dwell times of the other pH dependence experiment belonging to the high state (red) in seven pHs between 6.5 and 9.5 in Mn^{2+}: (a) 6.5, (b) 7.0, (c) 7.5, (d) 8.0, (e) 8.5, (f) 9.0, and (g) 9.5.

Section 7. Theoretical calculations

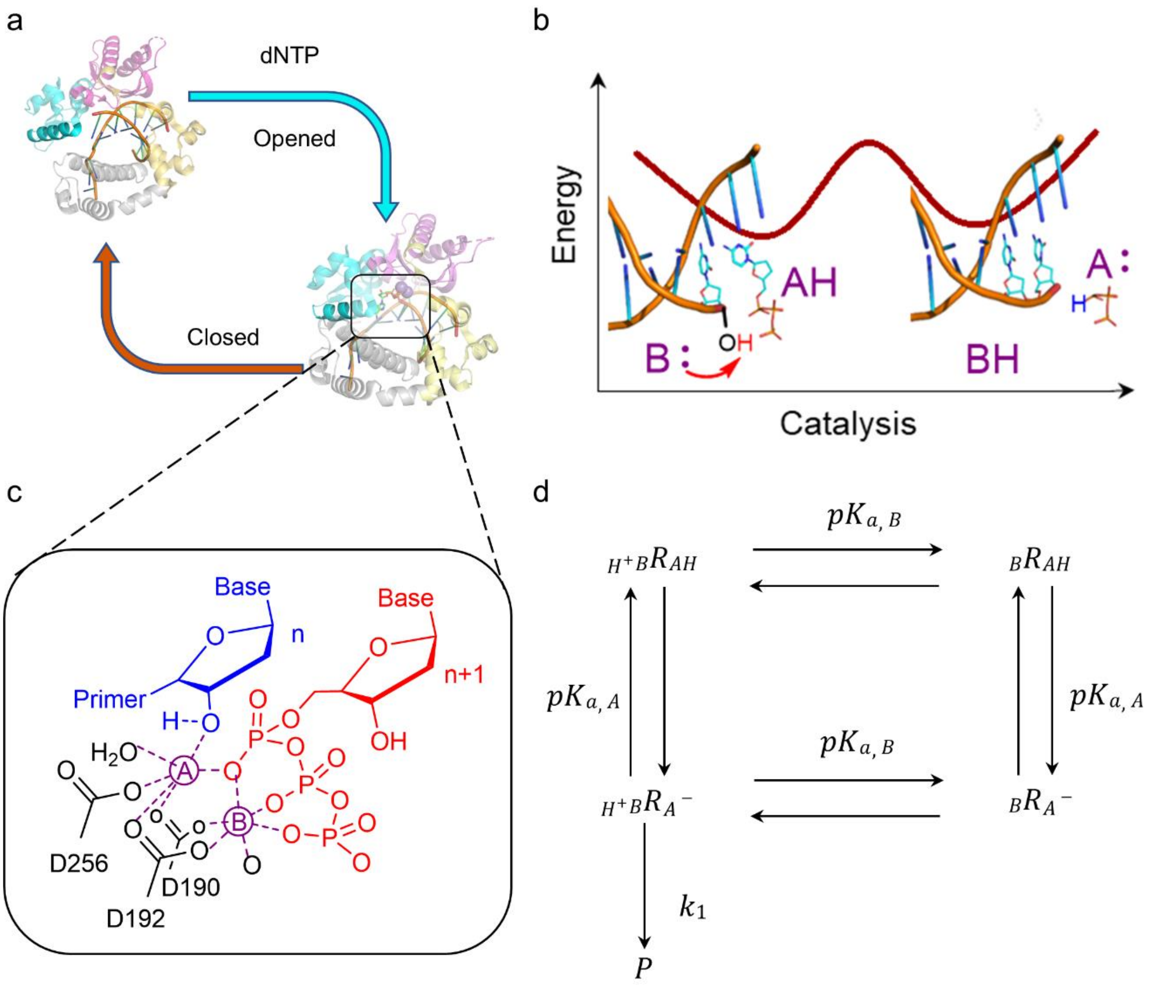

Figure S34. Nucleotide addition cycle of hPol β. (a) A conformational change of hPol β in each catalytic cycle. The active site is marked by a rectangle. (b) The active site. hPol β has three additional conserved acidic residues (gray) coordinated exclusively to the A-site and B-site metals. In the chemical step, the 3′-OH group of the primer terminus (blue) is deprotonated and attacks the P_{α} atom of the incoming nucleotide (red). (c) A simplified general base and a general acid model in hPol β-catalyzed nucleotidyl transfer reactions. (d) Double-Deprotonation Mechanism.

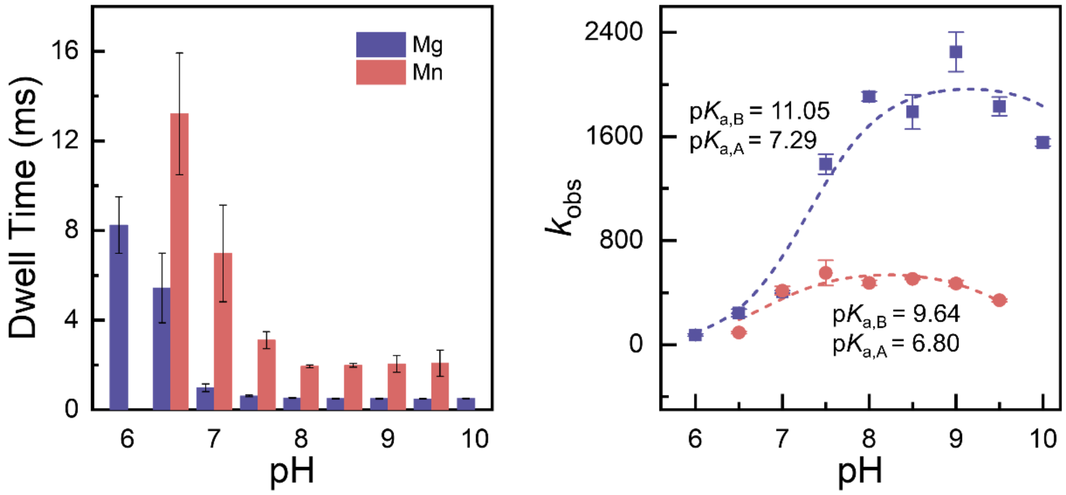

Figure S35. Summary of the pH dependence for nucleotidyl-transfer reaction (from Figure S23 and Figure S24), yielding pK_a values of about 11.05 for Mg2+ (filled squares) and about 9.64 for Mn2+ (filled circles).

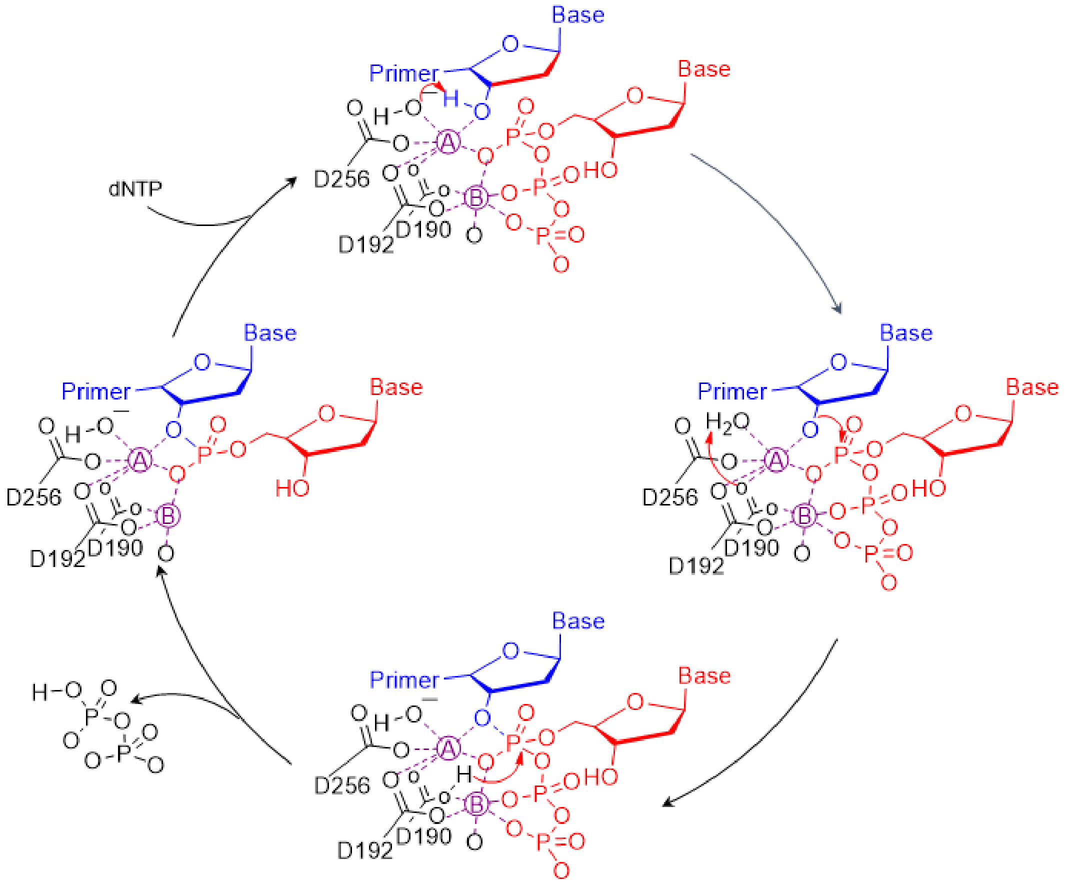

Figure S36. Catalytic mechanism of the catalytic cycle of hPol *β*.

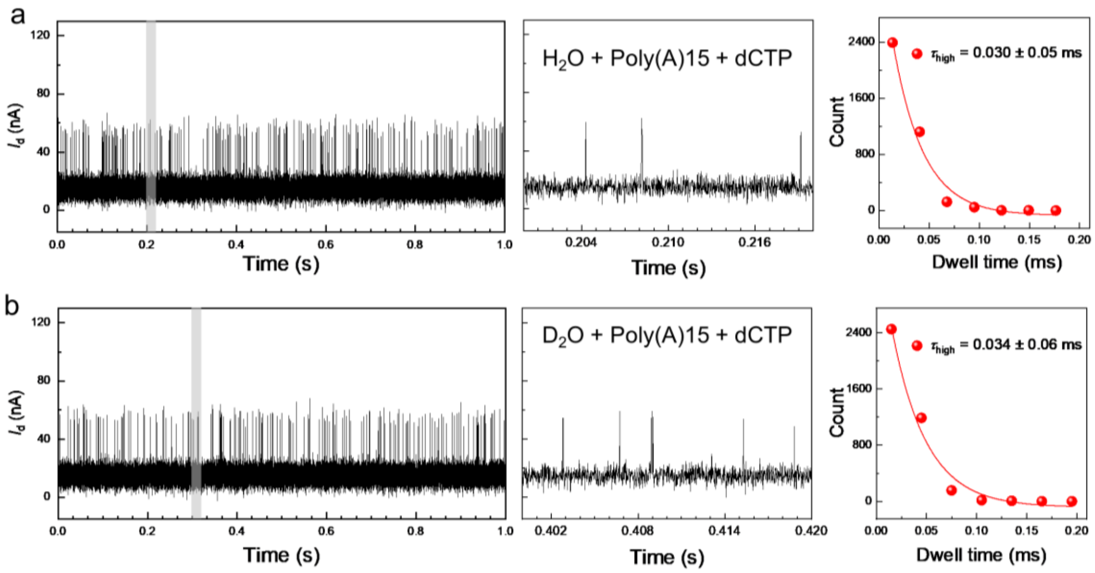

Figure S37. Characterization of nucleotide binding in mismatched substrates. (a) In H_2O buffer solution. (b) In D_2O buffer solution. In this experiment, Poly(A)$_{15}$ serves as the template, with dCTP acting as the substrate. *I*–*t* curves for 1 s (Left). Enlarged display for 0.02 s (middle). The dwell time distributions (right).

Moreover, in the exploration of potential applications by utilizing this platform to characterize nucleotide binding in mismatched substrates, we have discovered that the dwell time (τ_{short}) of nucleotide binding in mismatched substrates does not display isotope effects and is short (Figure S37). The results of the experiments also proved that the low-pass-filtered binary switch cut-off signal corresponds to mismatched nucleotide binding and thus the low pass filtered signal corresponds to the relatively slow matched nucleotide insertion reaction.

Section 8. The equation for the formation of COO^{-}–1/2Mg^{2+}

The hydrogen ion in the D190 side group (carboxyl group) was replaced by Mg^{2+}. Then a carboxylic acid-Mg structure (COO^{-}–1/2Mg^{2+}) was formed, which disturbed the original p*K*a~3.65 of the amino acid side group to neutral.[7] The COO^{-}–1/2Mg^{2+} had the following two equilibria:

$$R-COO^{-}+H_2O \rightleftharpoons R-COOH+OH^{-}$$

$$Mg^{2+}+H_20 \rightleftharpoons Mg(OH)^{+}+H^{+}$$

The result is that COO^{-}–1/2Mg^{2+} exhibits a p*K*a at 7.88.

Section 9. Sequence of the DNA hPol *β* and DNA template

Table S2. Complete sequence of the DNA hPol *β*

Before mutation

mhhhhhhlgg ggs +

1 m s k r k a p q e t l n g g i t d m l t e l a n f e k n v s q a i h k y n a y r k a a s v i a

61	k y p h k i k s g a	e a k k l p g v g t	k i a e k i d e f l	a t g k l r k l e k	i r q d d t s s s i	n f l t r v s g i g
121	p s a a r k f v d e	g i k t l e d l r k	n e d k l n h h q r	i g l k y f g d f e	k r i p r e e m l q	m q d i v l n e v k
181	k v d s e y i a t v	c g s f r r g a e s	s g d m d v l l t h	p s f t s e s t k	q p k l l h q v v e	q l q k v h f i t d
241	t l s k g e t k f m	g v c q l p s k n d	e k e y p h r r i d	i r l i p k d q y y	c g v l y f t g s d	i f n k n m r a h a
301	l e k g f t i n e y	t i r p l g v t g	v a g e p l p v d s	e k d i f d y i q w	k y r e p k d r s e	

After mutation

mhhhhhhlgg ggs +

1	m s k r k a p	q e t l n g g i t d	m l t e l a n f e k	n v s q a i h k y n	a y r k a a s v i a	
61	k y p h k i k s g a	e a k k l p g v g t	k i a e k i d e f l	a t g k l r k l e k	i r q d d t s s s i	n f l t r v s g i g
121	p s a a r k f v d e	g i k t l e d l r k	n e d k l n h h q r	i g l k y f g d f e	k r i p r e e m l q	m q d i v l n e v k
181	k v d s e y i a t v	c g s f r r g a e s	s g d m d v l l t h	p s f t s e s t k	q p k l l h q v v e	q l q k v h f i t d
241	t l s k g e t k f m	g v c q l p s k n d	e k e y p h r r i d	i r l i p k d q y y	c g v l y f t g s d	i f n k n m r a h a
301	l e k g f t i n e y	t i r p l g v t g	v a g e p l p v d s	e k d i f d y i q c	k y r e p k d r s e	

Table S3. DNA template

3’ –ACATTTTGCTGCCGGTCA AAAAA AAAAA AAAAA–5'

5’ –TGTAAAACGACGGCCAGT–3'

References

[1] Liu, W., Li, J., Xu, Y., Yin, D., Zhu, X., Fu, H., Su, X., Guo, X., *Adv. Sci.* **2021,** *8,* 2101383.

[2] Venkatasubban, K.S., Schowen, R.L., *CRC Crit. Rev. Biochem.* **1984,** *17,* 1–44.

[3] Shah, A. M.; Li, S.–X.; Anderson, K. S.; Sweasy, J. B., *J. Biol. Chem.* **2001,** *276,* 10824–10831.

[4] Alberts, I. L., Wang, Y., Schlick, T., *J. Am. Chem. Soc.* **2007,** *129,* 11100–11110.

[5] O'Flaherty D.K., Guengerich F.P., *Curr. Protoc. Nucleic. Acid. Chem.* **2014,** *59,* 7–21.

[6] Raper, A. T., Reed, A. J., Suo, Z., *Chem. Rev.* **2018,** *118,* 6000–6025.

[7] Bevilacqua, P. C. J. B., *Biochemistry* **2003,** *42,* 2259–2265.
